# Universal patterns in passenger flight departure delays

**DOI:** 10.1038/s41598-020-62871-6

**Published:** 2020-04-23

**Authors:** Yanjun Wang, Yakun Cao, Chenping Zhu, Fan Wu, Minghua Hu, Vu Duong, Michael Watkins, Baruch Barzel, H. Eugene Stanley

**Affiliations:** 1 Nanjing University of Aeronautics and Astronautics, College of Civil Aviation, Nanjing, 211106 China; 2 Massachusetts Institute of Technology, Department of Aeronautics and Astronautics, Cambridge, 02139 United States; 3 Nanjing University of Aeronautics and Astronautics, Department of Applied Physics, Nanjing, 211106 China; 4 Air Traffic Management Bureau of Northwest China, Air Traffic Control Division, Xi’an, 710000 China; 5 Nanyang Technological University, Air Traffic Management Research Institute, Singapore, 637460 Singapore; 6 Federal Aviation Administration Asia/Pacific Office, Singapore, 258508 Singapore; 7 Bar Ilan University, Mathematics Department, Ramat Gan, 5290002 Israel; 8 Boston University, Center for Polymer Studies and Department of Physics, Boston, MA 02215 United States

**Keywords:** Civil engineering, Civil engineering, Statistical physics, thermodynamics and nonlinear dynamics, Statistical physics, thermodynamics and nonlinear dynamics

## Abstract

Departure delays are a major cause of economic loss and inefficiency in the growing industry of passenger flights. A departure delay of a current flight is inevitably affected by the late arrival of the flight immediately preceding it with the same aircraft. We seek to understand the mechanisms of such propagated delays, and to obtain universal metrics by which to evaluate an airline’s operational effectiveness in delay alleviation. Here we use big data collected by the American Bureau of Transportation Statistics to design models of flight delays. Offering two dynamic models of delay propagation, we divided all carriers into two groups exhibiting a shifted power law or an exponentially truncated shifted power law delay distribution, revealing two universal delay propagation classes. Three model parameters, extracted directly from dual data mining, help characterize each airline’s operational efficiency in delay mitigation. Therefore, our modeling framework provides the crucially lacking evaluation indicators for airlines, potentially contributing to the mitigation of future departure delays.

## Introduction

Millions of passengers worldwide routinely suffer from flight delays, which not only constitute a common inconvenience, but are also a source of tremendous economic loss and potential chaos in air traffic. To further exacerbate the issue, the interconnected nature of airports and carriers, where the arrival of planes and crews depends on the the punctuality of incoming and outgoing flights, leads to the potential spread of delays from one destination to another, in comparison with information spreading^1–3^. Hence, to alleviate the impact of such delays, we seek to understand the underlying rules that govern their propagation by uncovering their recurring statistical patterns^4,5^. This is made possible thanks to the collection of big data^6–9^ on flights, leading in recent years to significant advances in our understanding of the delay dynamics^5,10–19^. Still, most current analyses are limited to just one or few airlines, or to a restricted time-span, limiting the scope of their potential insight, and inevitably overlooking universal patterns that can only be observed by comparisons across airlines or over many years. Here we use, for the first time, a comprehensive dataset, capturing flight records extracted from 14 United States carriers over the course of 20 years. Focusing on such a broad dataset, we are able to observe universal statistical patterns across different carriers, providing us with direct insight into their operational functionality towards delay mitigation.

The US Bureau of Transportation Statistics (BTS) classifies the causes of passenger flight delays into five categories^20^: (i) The carrier; (ii) extreme weather; (iii) the national aviation system; (iv) security; (v) late-arriving aircrafts; where category (v) is the factor directly causing *propagated delay* (PD) in which the departure delay of the current flight is inevitably caused by the late arrival of the immediately preceding flight with the same aircraft. We call it the PD factor, while we call categories (i) to (iv) non-propagation factors. Such downstream delay propagation^5,10–16,21–26^ begins with a last departure delay that may result from any one or more in the five causes (i) - (v), but then leads to an ensuing delay via PD, *i.e*. specifically cause (v). To understand the rules of this propagation we obtain the functional link between an immediately preceding delay and the resulting current PD with the same aircraft, namely we find out the probability density for a delay to propagate. We find that the observed propagation rules across the 14 carriers condense around two distinct functional forms. Together, they provide a direct insight into the operational efficiency of the airlines in mitigating and absorbing the potential propagation of departure delays.

## Results

**Figure 1:**
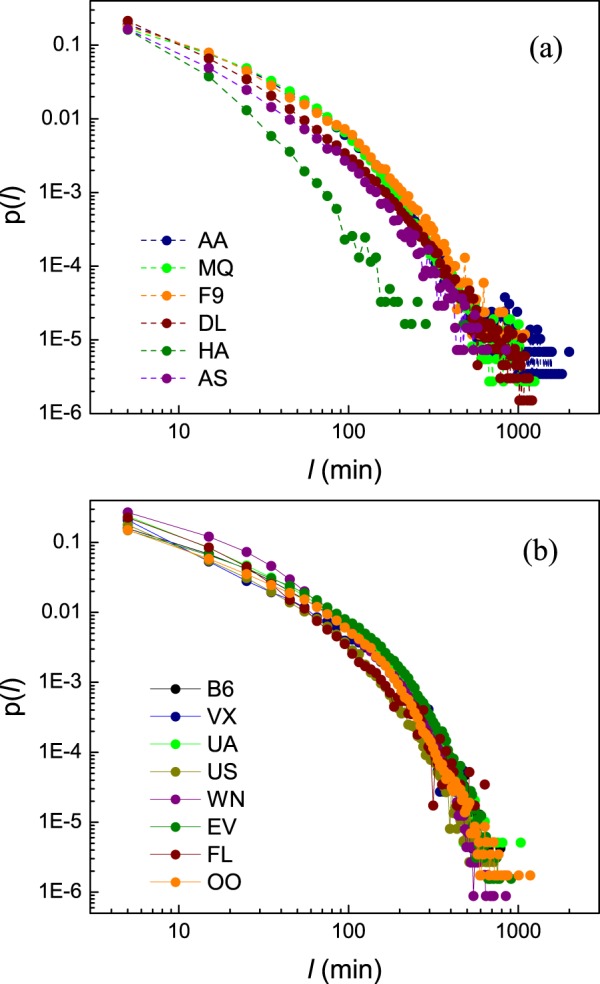
The probability density *p*(*l*) to observe the departure delays of length *l* per delay interval near *l*, obtained from the BTS data collected for 14 US airlines in 2014. Airlines are divided into two groups: (**a**) 6 airlines, AA, MQ, F9, DL, HA and AS, exhibit a shift power law distribution; (**b**) the remaining 8 airlines, B6, VX, UA, US, WN, EV, FL and OO, exhibit an exponentially truncated shift power law distribution. Time intervals are set to Δ*l* = 10 min.

We collected data on flight delays from the BTS^20^, as detailed in Table S1 of the Supporting Material (*SM*), together, covering the activity of 14 US carriers over a period of 20 years. To quantify departure delays we calculate the difference *l* (minutes) between the actual versus the scheduled departure time of each flight, hence the greater is *l* the more severe is the delay. We characterize the delay patterns through the probability density *p*(*l*) to observe a delay of magnitude *l*, as shown in Fig. 1. We find that the 14 airlines condense around two distinct Groups, 1 and 2. To observe this we present in Figs. 3A and 4A the complementary cumulative distribution functions (CCDF)^27–29^.

1 $${P}({{l}}_{0} > {l})={\int }_{l}^{\infty }{p}({l}){dl},$$

which capture the probability to observe a delay in excess of duration *l*. *Group* 1 (Fig. 3A). In 6 of the airlines (AA, MQ, F9, DL, HA, AS) the CCDF is best captured by a shift power law (SPL)^30–32^ of the form *P*(*l*_0_ > *l*):(*l* + *β*)^−*α*^. *Group* 2 (Fig. 4A). For the remaining 8 airlines we observe an exponentially truncated shift power law (ETSPL)^33^, in which the tail exhibits an exponential cutoff. Below we show that these two classes represent two distinct models of propagated delays, providing a window into the airlines’ operational efficiency in delay mitigation.

**Figure 2:**
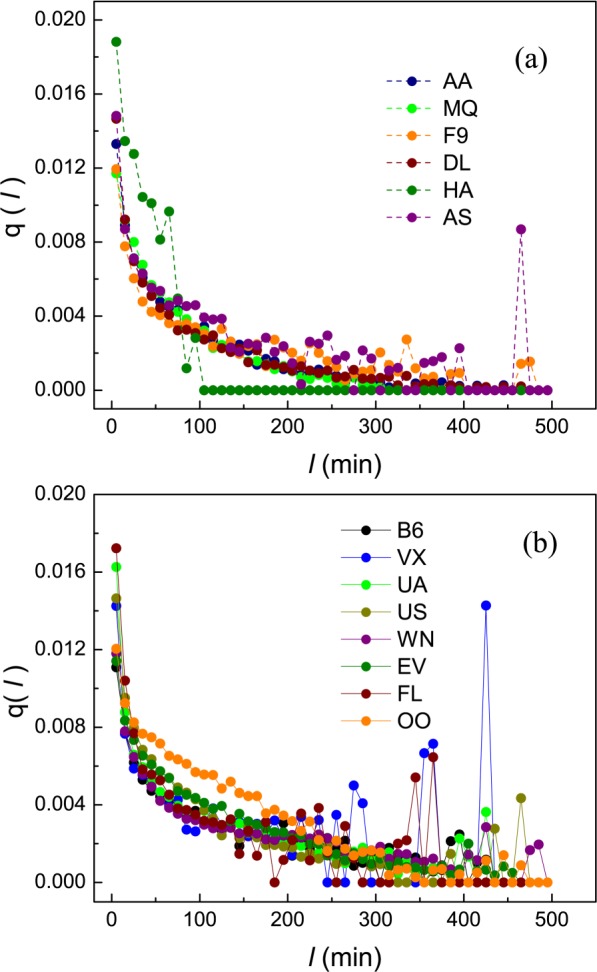
The transfer density function *q*(*l*) vs. *l* obtained from the empirical BTS data. This function captured the intensity of all the previous departure delays that propagated to cause a DTPD. Data covers the statistics from Year 2014’s primary records over the 14 US airlines. (**a**) Airlines AA, MQ, F9, DL, HA and AS in Group 1; (**b**) Airlines B6, VX, UA, US, WN, EV, FL and OO of Group 2. Δ*l* = 10 min.

**Figure 3:**
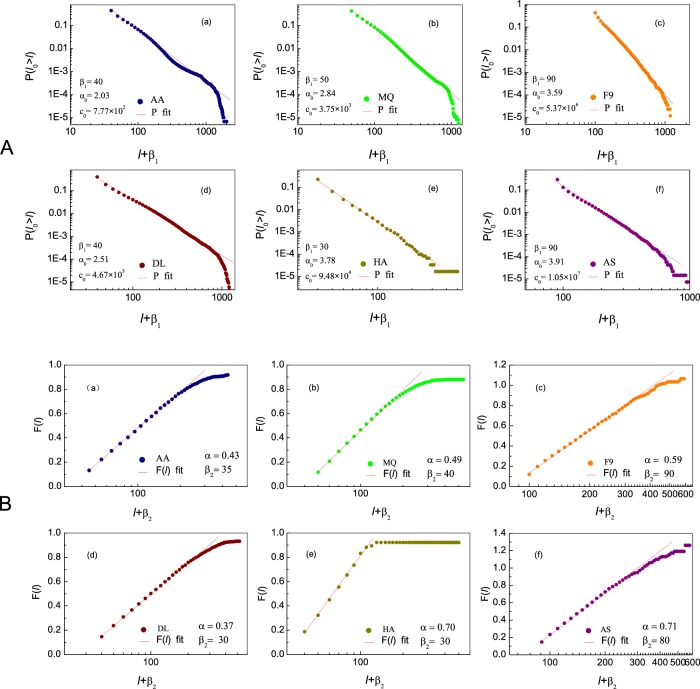
(**A**) The complementary cumulative distribution functions (CCDFs) *P*(*l*_0_ > *l*) of empirical data extracted for Group 1 (airlines AA, MQ, F9, DL, HA and AS (filled colored circles). Fitting to each of them with a shifted power law in the form of Eq. (9), we obtain parameters *β*, *α*_0_ and *c*_0_. We denote such *β* by *β*_1_ in each panel. (**B**) The empirically obtained *F*(*l*) from Eq. (10) vs. the shifted time interval *l* + *β*_2_ based on Model 1 for the 6 airlines (circles). The shift parameter *β*_2_ is tuned to obtain the best fit of *F*(*l*) with the integrated *q*(*l*), as taken from Eq. (4) (solid red lines). The resulting estimator *β*_2_ appears in each panel. In all panels we used time intervals of Δ*l* = 10 min.

**Figure 4:**
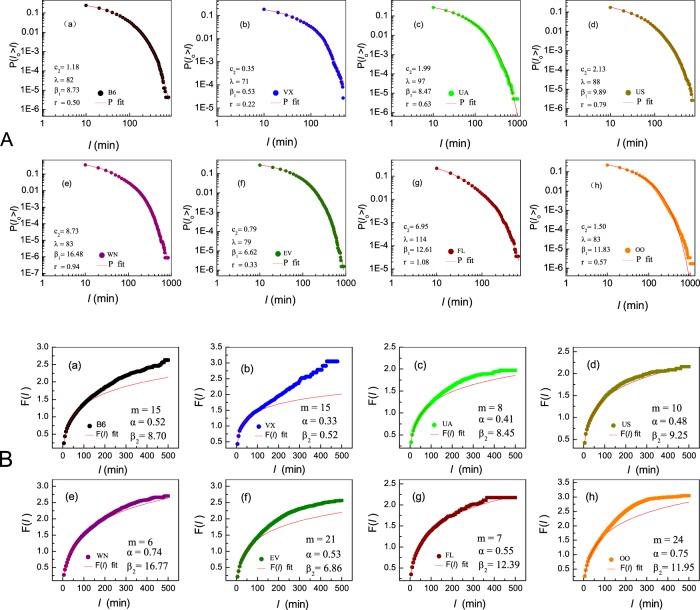
(**A**) The CCDF *P*(*l*_*0*_ > *l*) as obtained from empirical data in Group 2 (8 airlines: B6, VX, UA, US, WN, BV, FL and OO) (filled colored circles). Fitting (13) (red solid lines) to them, we extract the fitted parameters *c*_2_, *λ*, *β*_1_ and *r* (listed in each panel). (**B**) *F*(*l*) vs. *l* as obtained from empirical data (circles) based on Eqs. (2) and (10). To obtain the compensation interval *m* we fit the observed *F*(*l*) with the the integrated *q*(*l*) with compensation *m* (solid red lines). By appropriately tuning *m* we obtain the best fit in which the estimator *β*_2_ approaches its CCDF-based counterpart *β*_2_. In all panels we set Δ*l* = 5 min.

### Decreased type of propagated delay

The effects of propagation are not readily discernible from the primary data^34^. On the one hand, given two successive delayed flights carried by the same aircraft, delay time of a current departure may be not merely attributed to the late arrival of the flight immediately preceding it, but also be attributed to one or more other factors, *e.g*., extreme weather on arrival airport. On the other hand, the role of non-propagation factors is likely diminished in the cases where the current departure delay is shorter than the one immediately preceding it with the same aircraft. This decrease of delay time expresses the efforts of the airline’s operational staff in countering PD, hence characterizing the carrier’s ability to absorb delays. Therefore, we concentrate below on decreasing type of propagated delays (DTPD), in which a propagated delay of an aircraft at *j* was shorter than its immediately preceding one at *i*.

### Model 1 — Flight delay distributions with shift power law

The conception of a PD is rooted in an immediately preceding departure delay, caused by any of the aforementioned reasons, from category (i) to (v), which then propagates to cause a current delay *l* with the same aircraft. In the case of DTDP, the initial delay can be of any length longer than *l*, here we seek the probability per delay interval near *l*, *q*(*l*), that a delay in the range (*l*, ∞) propagates to cause a delay of length *l*. Denoting by *n*_*B*_(*l*) the total number of DTPD instances per delay interval near *l*, we write

2 $${{n}}_{{B}}({l})={q}({l}){N}({l}),$$

where

3 $$N(l)={\int }_{{l}}^{\infty }n(l)\varDelta l$$

represents the total number of departure delays with a lag greater than *l*; the delay number density *n*(*l*) is the number of delays per delay interval near *l*. The transfer function *q*(*l*) captures the probability density for longer delays with number *N*(*l*) to generate a DTPD - hence providing the contribution of *N*(*l*) that transfers into *n*_*B*_(*l*). The reasoning behind Eq. (2) is analogous to the classic mean-field approach of statistical physics, where current delays are assumed to be affected by all previous delays, via the *l*-dependent transfer density function *q*(*l*). We obtain the density function *q*(*l*) directly from data mining, by comparing the ratio of DTPD number density *n*_*B*_(*l*) and the total number of delays *N*(*l*) with the delayed intervals longer than *l*. We find that the probability of a flight having a delay *l* by DTPD decreases with *l* (Fig. 2), and therefore the observed transfer density functions *q*(*l*) can be effectively approximated by

4 $${q}({l})=\frac{{\alpha }}{{l}{+}{\beta }},$$

an inverse linear function, in which the parameters *α* and *β* represent airline - specific phenomenological parameters.

Next, we assume that DTPD represents a constant fraction *k* of all delays, allowing us to write

5 $${{n}}_{{B}}({l})={kn}({l}).$$

Substituting (5) into Eq. (2) we obtain

6 $${kn}({l})={q}({l}){N}({l}),$$

which using (3), or alternatively, $n(l)=\frac{N(l)-N(l+dl)}{dl}=-dN/dl$ since *N*(*l*) is proportional to CCDF *P*(*l*_0_ > *l*), provides us with

7 $$\frac{{dN}}{{dl}}=-\frac{{\alpha }}{{k}({l}+{\beta })}{N}({l}),$$

where we used (4) to express *q*(*l*). We, therefore, obtain

8 $${N}({l})={c}{({l}+{\beta })}^{-{{\alpha }}_{0}},$$

where *α*_0_ = *α*/*k*, and *c* is an integration constant. As a result, the analytical CCDF of departure delays is found to follow

9 $${P}({{l}}_{0} > {l})=\frac{{N}({l})}{{{N}}_{{0}}}={{c}}_{0}{({l}+{\beta })}^{-{{\alpha }}_{0}},$$

where *N*_0_ is the yearly total number of flights of an airline, and the constant *c*_0_ is defined as *c*_0_ = *c*/*N*_0_ (see Table S1 in the *SM*). This, indeed, represents the SPL^30–32^ form of *P*(*l*) observed in Fig. 1a for the six airlines in group one.

To further examine the relevance of Model 1 above, in Fig. 3A we fit the CCDFs(red lines) from Eq. (9) to the real data from BTS (colored filled circles) for the six airlines (Group 1) in Fig. 3A (for Year 2014). At least in three decades, in the form of shift power-law, red lines fit well to empirical data which are independent of Model 1. As the result, a unique pair of parameters *β* and *α*_0_ are obtained for each airline. Hence, while the form of the CCDF is universal, across all six airlines, the fitted parameters *β* and *α*_0_ represent airline specific phenomenological values, whose meaning we discuss below. Results of the remaining 19 years of data are shown in Figs. S1–S10 of *SM*.

The relevance of our assumptions, as expressed in Eqs. (2–5), can be reexamined by refitting the parameter *β*_2_, this time from the transfer density function *q*(*l*). This will provide us with two fitted evaluations of *β*: *β*_1_ extracted above from the CCDF, *i.e*., integrated *p*(*l*) of Fig. 1, and *β*_2_ extracted from Eq. (2). Their consistency, as well as any discrepancies between these two fits, can gain us an additional validation and insight pertaining to our model analysis. Hence, in Fig. 3B we present the integrated function .. versus the shifted delays *l* + *β*_2_ for all six airlines classified in model 1. Using Eq. (4) we write

10 $${F}({l})={\alpha }\text{ln}\left(\frac{{l}+{{\beta }}_{2}}{{{\beta }}_{2}}\right),$$

allowing us to extract *β*_2_ directly from the data, and thus yielding the desired independent evaluation of *β* in Eqs. (4) and (9). The obtained values for *β*_2_ appear in Fig. 3B, showing similar trends to the *β*_1_ values obtained from fitting Eq. (9) (red lines) to real data (colored filled circles) in Fig. 3A. Results of the remaining 19 years are shown in Figs. S30–S39 in the *SM*. These further support the relevance of our model and their analyses to the PD statistics.

When we fit Eq. (9) to practical distributions CCDFs, *i.e*., *P*(*l*_0_ > *l*) integrated from *p*(*l*) of Fig. 1a, we are calibrating the contributions from all five categories of delaying factors into DTPD assumed by Eq. (2), which yields parameters (*α*_1_, *β*_1_) for each airline (shown in Table S2 of *SM* for year 2014). On the other hand, *β*_2_, is extracted from the transfer density function *q*(*l*) capturing only the propagation instances that had a decreasing effect with *l*, *i.e*. DTPD. Such decreases in delay time are likely the results of all efforts from airlines, airports, air controllers, and so on. to overcome the PD. Hence the ratio of these two estimators, *β*_2_/*β*_1_, can help us characterize an airline’s operational effectiveness, quantifying how effectively that airline absorbs delays and attenuates their propagation.

Taken together, Model 1 is validated in Fig. 3 upon approximately three orders of magnitude, covering delay scales over several hundreds of minutes. Beyond that, all empirical curves (colored symbols) are truncated, deviating from the CCDF of Eq. (9) (red lines), indicating that the effects of non-propagation factors begin to dominate the delay dynamics. The empirically obtained *F*(*l*) curves saturate in the limit of large *l*, as *q*(*l*) approaches zero. In such cases, *F*(*l*) begins to deviate from the Eq. (10), as our approximate assumptions in Eq. (2) no longer hold.

### Model 2 — Flight delay distributions with exponentially truncated shift power law

We now turn to analyze the remaining 8 airlines (Group 2), whose CCDF features an exponential truncation in the limit *l* → ∞. Such behavior can arise from Eq. (7) if we assume the appropriate dependence of *k* on *l*, *i.e*. substitute the constant *k* by *k*(*l*). Such generalization of Model 1 relaxes the assumption that decreasing type of propagation delays (DTPD) occupy a constant fraction *k* of whole numbers of delays, and rather introduces an *l*-dependence, where, *e.g*., longer delays are less likely to be of the DTPD. Hence, we now replace *k* in Eq. (7) by

11 $${k}({l})=\frac{{1}}{{gl}+{h}},$$

which approaches a constant (1/*h*) in the limit of small *l*, but tends to zero as *l* → ∞. Model 2, therefore, converges to Model 1 in the limit where *g* is vanishingly small. As above, the parameters *g* and *h* represent phenomenological constants, extracted for each airline from the data. Using (11) in (7), we obtain

12 $${N}({l})={{c}}_{{1}}{{e}}^{-\frac{{l}}{{\lambda }}}{({l}+{\beta })}^{-{r}},$$

where *λ* = 1/*gα*, *r* = *h* − *αβg* and *c*_1_ is an integration constant. Consequently, the CCDF of departure delays now takes the form

13 $${P}({{l}}_{0} > {l})=\frac{{N}({l})}{{{N}}_{0}}={{c}}_{2}{{e}}^{-\frac{{l}}{{\lambda }}}{({l}+{\beta })}^{-{r}}$$

where *c*_2_ = *c*_1_/*N*_0_. Unlike the SPL of Eq. (9), the 8 airlines in group 2 follow an exponentially truncated shift power law (ETSPL)^33^, as exactly we observe in Fig. 4A. The CCDF in Eq. (13) is characterized by four independent parameters, *c*_2_, *λ*, *β* and *r*, that can all be obtained by fitting Eq. (9) to the empirical distributions, as we show in Fig. 4A with data obtained from Year 2014 (see also Table S2 in *SM*), since CCDF obtained from data mining are independent of any model. Results of the remaining 19 years are shown in Figs. S11–S29 of *SM*.

### Performance metrics for delay mitigation

Our analysis allows us to extract three empirically accessible parameters with direct relation to an airline’s treatment of PD.

#### Compensation parameter m

Consider the parameter *β* in (4). We can evaluate it through two independent empirical functions: the CCDF in (13), as done in Fig. 4A, yielding the estimator *β*_1_; or, alternatively, the cumulative function *F*(l) in (10), providing the estimator *β*_2_ (Fig. 4B). While both are aimed at evaluating the same parameter *β*, these two estimators are naturally inconsistent. In the CCDF, since we cannot distinguish the effects of these two kinds of delay factors^34^, all types of delays, including even cancelations^35,36^, are intermixed. In contrast, *F*(*l*), based on *q*(*l*) from Eq. (2), is constructed exclusively from DTPD. Therefore, we expect an inevitable discrepancy between the two estimates *β*_1_ and *β*_2_. To correct for this discrepancy we re-evaluate *F*(*l*), to include the impact of increasing type of propagation delays (ITPD), thus bringing it closer to the observed CCDF. We achieve this by redefining *n*_*B*_(*l*) in Eq. (2) to include also contributions of ITPD, namely it now counts the number density of flights with delay around *l*, whose immediately preceding delay *l*′ ≥ *l* − *m*. The compensation parameter *m* allows us to include ITPD and impacts of non - propagation factors in our PD analysis. Therefore, in the limit *m* → 0, *n*_*B*_(*l*) is restricted to just DTPD, *i.e. l*′ ≥ *l*, reverting back to its original definition in Eq. (2). However, for a larger *m*, *n*_*B*_(*l*), and hence also *q*(*l*) and *F*(*l*), account for a growing contribution of ITPD. By tuning the parameter *m* we can now *force* the estimated *β*_2_ to become consistent with *β*_1_, as we demonstrate in Fig. 4B. For example, for airline B6 we set *m* = 15 to reach *β*_2_ = 8.70, a value that is within 0.3% of the estimated *β*_2_, which is evaluated at 8.73; Fig. 4A(a),B(a).

The parameter *m* characterizes the impact of ITPD together with that of DTPD in shaping the delay distribution *P*(*l*). Indeed, a small *m* indicates a dominant role of DTPD, while for a larger *m*, a greater factor of ITPD is introduced in order to reconcile *F*(*l*) with *P*(*l*_0_ > *l*). Therefore, large *m* characterizes an airline in which ITPD plays a significant role, that is to say, a poor performance in mitigating PD. Consequently, *m* offers an inverse metric of the performance to evaluate an airline’s success in absorbing delays and attenuating their propagating impact.

#### shift parameter β

**Figure 5:**
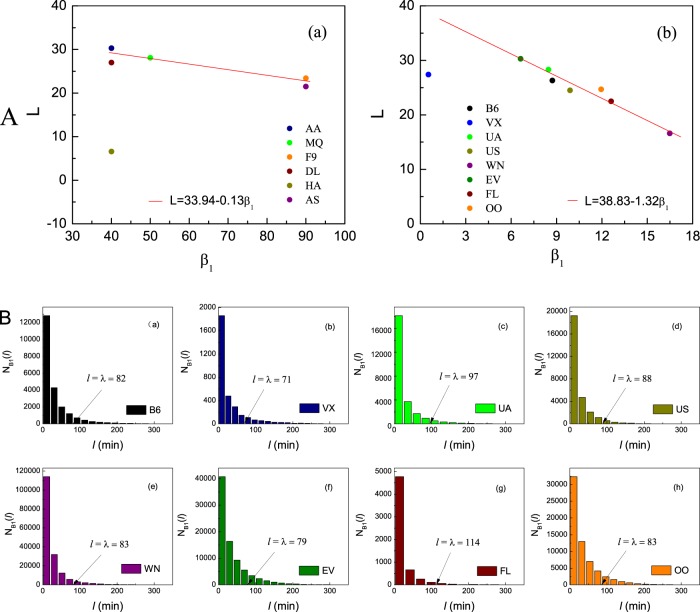
(**A**) (a) The average absorption time *L* vs. the estimated shift parameter *β*_1_ as obtained from empirical data pertaining to the 6 Model 1 airlines (circles). We observe a linear relationship of the form (14) with a slope of *K* = −0.13 (solid red line). Airline HA is excluded from the fit, due to its distinctive operation style, as compared to the other five airlines (see main text). (b) *L* vs. *β*_1_ for the 8 Model 2 airlines, exhibiting a slope of *K* = −1.32. Here VX is an outlier, likely due to the fact that it has fewer data points compared to all other airlines. (**B**) Number distributions *N*_*B*1_(*l*) of delayed flights in 8 airlines are biased to the side with *l* < *λ*. Histograms are assigned with equal width $\varDelta l=\frac{1}{4}\lambda$. Arrows indicate the positions of critical value *λ* separating the dominant range of the propagation factor from those of ITPD and non - propagation factors for airlines B6, VX, UA, US, WN, EV, FL and OO, respectively. The functions *N*_*B*1_(*l*) means the delayed flight number caused by the propagation factor based on direct counting of flights with longer delays of immediately preceding flights than the current ones.

A crucial quantity in the context of DTPD is the average flight delay absorption time *L* = *l*_*p*_ − *l*_*c*_ (minutes), capturing the level of decrease in delay time between the preceding departure delay (*l*_*p*_) and the current propagated delay (*l*_*c*_) with the same aircraft^37,38^. A successful delay mitigation is captured by a large *L*, *i.e*. a significant decrease in delay from preceding to current flight. We find, from the data, that *L* can be directly related to the shift parameter *β*_1_ through an approximate linear relationship (Fig. 5A)

14 $${L}({{\beta }}_{1})={Q}+{K}{{\beta }}_{1},$$

capturing a negative correlation between *β*_1_ and *L*. For the 6 airlines within Group 1, we observe that *K* ≈ −0.13(*Q* = 33.94), while for the 8 airlines of Group 2 we estimate *K* ≈ −1.32(*Q* = 33.83), roughly an order of magnitude difference. Therefore, on average, the smaller *β*_1_ is, the greater *L* is, and hence the more effective the airline in absorbing delays is. In Fig. 5A we exclude two airlines from the linear fit: in panel (a) the carrier HA is a distinctive outlier. This is rooted in its unique *point-to-point* operation style which is different from the common *hub-and-spoke* style^39^. Therefore, HA has intrinsically different propagation patterns. In panel (b) VX has a smaller flight volume compared to all other carriers (see Table S1 in **SM**) and it, therefore, falls below the mainstream statistical line. Equation (14) based on Fig. 5A show that airlines with smaller values *β*_1_ are more effective in absorbing delays from the immediately preceding ones, which can help us to understand that the majority of flights with shorter delays (*l* < *λ* in Fig. 5B), where *n*_*B*_(*l*) in formula (1) are cumulated into histograms of *N*_*B*1_(*l*) $({{N}}_{{B}1}({l})={\int }_{{{l}}_{1}}^{{{l}}_{2}}{{n}}_{{B}}({l}{{\prime} }){d}{l}{{\prime} })$ with each width set as $\frac{1}{4}{\lambda }$, where *l* is the mean value of *l*_1_ and *l*_2_. Average absorption time *L* in Group 2 behaves more sensitive than those in Group 1, since *L* changes over about 10 minutes against the *β*_1_ variation over the range of 50 minutes in Fig. 5A(a) for Group 1, while *L* changes over about 15 minutes against the *β*_1_ variation over only about 11 minutes in Fig. 5A(b) for Group 2. The comparison is effective within each panel (a) and (b) of Fig. 5A, respectively. For instance, airline AA behaves more effective in absorbing the preceding delays than airline AS in Group 1; while EV behaves more effective in delay - absorption than WN in group 2, during 2014. Plots of *L*(*β*_1_) for airlines operated in other 19 years are shown in Figs. S59–S69 of *SM*.

#### Critical delay λ

Now we turn to the role played by parameter *λ*. It acts as the critical delay of airlines in Group 2. CCDFs by Eq. (13) fit empirical data well because they are obtained with the aid of one more phenomenological function *k*(*l*). However, practical *q*(*l*) mined from primary data goes to zero not in the manner assumed by Eq. (4). Actually, empirical *q*(*l*) often touches zero beyond 240 minutes and fluctuates more or less above it (see Fig. 2). Therefore, cumulated *q*(*l*), *i.e*., colored filled circles obtained from data mining shown in Fig. 4B drastically deviate from the integrated transfer density function *F*(*l*) (red lines) from Eq. (4) starting at the critical delays (*l* = *λ*)(see panels in Fig. 4B). The exact fitting between analytical and empirical *F*(*l*) in the range *l* < *λ* indicates that the assumption of DTPD is successful; while deviations shown in Fig. 4B mean that *F*(*l*) integrated from *q*(*l*) becomes less effective as *l* exceeds *λ*, indicating a gradually increasing impacts of ITPD and non - propagation factors, since DTPD becomes less dominant as *l* increases. Therefore, *λ* serves as the critical delay separating DTPD dominated range from the range dominated by above mentioned two others. Moreover, the growing error effect starting at the deviations (*l* = *λ*) can be estimated with Fig. 5B. The cumulated flight number *N*_*B*1_(*l*) often occupies the fraction larger than 90 percent as seen in it, manifesting successful degree of the assumption DTPD. Furthermore, we have intuitive results from the comparisons based on simple observations. Comparing panel(a) and (e) in Fig. 4B, also, taking the reference of values *λ* in counterparts of Fig. 4A, we see that red lines from Model 2 deviate more from real *F*(*l*) (filled colored circle) with larger *m* but similar values of *λ*; The same result was concluded for panel (d) and (f); While comparing panel (c) with (g),red line deviates more from real data for smaller *λ* but with almost equal values *m*; Comparing four central panels, ((b) and (f) with (c) and (g)), we see that red lines with both larger *λ* and smaller *m* from Model 2 fit better with real data of airlines in Group 2. Actually, the effect of the exponential factor in Eq. (13) becomes stronger as *l* grows beyond *λ*. The larger *λ* an airline has, the larger it has the dominant range of DTPD, which is the positive effect in delay mitigation. In this way, combining the observation of behaviours of *λ* and *m* in corresponding panels of both Fig. 4A,B, and the observation of the behaviors of them in other 19 years shown in Figs. S11–S29, and Figs. S40–S58, we capture an obvious tendency: the smaller *m* and the larger *λ* an airline has, the better for Model 2 to fit to the results mined from big data, and the better it operates, since DTPD governs larger range of delay interval (0, *λ*) supported by all efforts to counter the propagated delays.

## Discussion and Conclusions

### Discussion of techniques used

The mean-field approach in temporal regime has not been fully explored in previous works. Typically, the mean-field approach is used in the following setting: given a particle, the effect of interactions from all surrounding particles is represented instead by an equivalent external spatial field. In this paper, we use an analogous approach given in Eq. (9). Specifically, the delay (in *l* minutes) of a current flight is the result of delays transferred from all previous flights with delays longer than *l*. The phenomenological function *q*(*l*) acts to transfer intensity. In this way, we model the main stream tendency of air transportation as a DTPD. Note that both Model 1 and Model 2 have this main assumption. Moreover, an airline with a better model fit indicates a stronger tendency to be DTPD in terms of its operations, and thus its ability to mitigate departure delays. We observed this behavior through empirical flight delay data from the US.

We note that assumption 1 (Eqs. (2) and (3)) is only partially correct since we incorporated the impacts from four other categories (i–iv) as well as ITPD into that of the DTPD. However, we keep with this assumption because admits analytical derivations with good fits to real data. Moreover, we can distinguish airline-specific differences between *β*_1_ and *β*_2_, as well as the emergence of metrics *m* and *λ*. Specifically, this allows us to check the precision of our models and validation ranges. Note that the CCDFs are model-free; they are obtained purely from data.

### Conclusions

Using the large scale data from BTS of US, we exposed two universal patterns of propagated departure delays of passenger flights, separating airlines into two distinctive groups — one with an SPL and the other with ETSPL CCDF. These groups exhibit two different *mechanisms* of delay propagation, as captured by Model 1 and Model 2. In the majority of cases the airlines remained consistent along this divide, however, few exceptions, in which airlines changed classification over the years have been observed (EV, AS, DL)(see *SM*) – a phenomenon that deserves further investigation.

Our analysis, investigating the relative roles of DTPD vs. ITPD, identifies three parameters that can help characterize an airline’s operational performance in avoiding PD. The shift parameter *β*, which negatively correlates with the average delay absorption time *L*; the compensation interval *m* that quantifies the participation of ITPD as compared to DTPD; and the critical delay *λ* that separates the delay interval that is dominated by DTPD from that of ITPD and non-propagation factors. Together, they offer quantitative empirically accessible metrics for performance evaluation over time and across airlines.

Tracking the temporal behavior of these three metrics over time we can observe the impact of the US instituted Airline Passenger Protection rules (also known as Passenger Bill of Rights) in 2009, as well as their update in 2012^40^. These rules reduced the number of commercial aircraft delays in excess of 240 minutes, prompting the carriers to cancel such flights and reroute passengers. This is observed through the enhancement of operational performance exhibited by most airlines directly after 2008, and, again, after 2012 (Fig. S69 in *SM*). This not only indicates the positive impact of these US regulations instituted in 2009 and 2012, but also provides independent validation for the relevance of our proposed metrics, that were, indeed, able to detect this regulatory shift. Hence, we offer these three metrics as a basis for air transportation assessment in both the US and other countries.

In a broader perspective, we believe that our statistical physics inspired approach can advance our understanding of systems that are well beyond the realm of standard physical systems^41^. Indeed, focusing on statistical properties, and constructing simplified models, we were able to characterize a seemingly unpredictable and highly complex phenomenon, such as passenger flight delays, and expose its universally recurring and consistently predictable patterns.

## Supplementary information

Supplementary Material.
