# Supplementary material for: Universal patterns in passenger flight departure delays
Source: Sci Rep. 2020 Apr 23;10:6890. doi: 10.1038/s41598-020-62871-6 (PMC7181864; doi:10.1038/s41598-020-62871-6)
Supplement: Supplementary file 1 — Supplementary Material. [file 41598_2020_62871_MOESM1_ESM.pdf]

Supplementary Material – Universal patterns in passenger flight departure delays

Yanjun Wang,[1,2,*] Yakun Cao,[3,*] Chenping Zhu,[3,†] Fan Wu,[1,4] Minghua Hu,[1]
Vu Duong,[5] Michael Watkins,[6] Baruch Barzel,[7] and H. Eugene Stanley[8,†]

[1]*College of Civil Aviation, Nanjing University of Aeronautics and Astronautics, Nanjing 210016, China*
[2]*Department of Aeronautics and Astronautics, Massachusetts Institute of Technology, Cambridge, MA 02139, United States*
[3]*Department of Applied Physics, Nanjing University of Aeronautics and Astronautics, Nanjing 210016, China*
[4]*Air Traffic Management Bureau of Northwest China, Air Traffic Control Division, Xi'an, 710000, China*
[5]*Air Traffic Management Research Institute, Nanyang Technological University,Singapore 637460, Singapore*
[6]*Federal Aviation Administration Asia/Pacific Office, Singapore 258508, Singapore*
[7]*Mathematics Department, Bar Ilan University, Ramat Gan 5290002, Israel*
[8]*Center for Polymer Studies and Department of Physics, Boston University, Boston, Massachusetts 02215, USA*

(Dated: December 10, 2019)

GENERAL MESSAGE OF 14 MAIN AIRLINES IN 2014

Tables of operation message of domestic passenger flights of 14 airlines in 2014

TABLE S1. General message of 14 airlines before and after the filtering with the threshold of 1000 flights per year for an aircraft in 2014

airlines	ave.flights/day*aircraft	No.aircrafts	No.aircrafts with No. flights > 1000	Total No.flights aft. Filtering
AA	3.4	676	237	291510
MQ	4.6	224	217	367189
F9	4.1	56	54	84349
DL	3.8	783	475	662197
HA	9.3	49	18	60946
AS	3.7	142	112	154600
EV	4.4	416	401	643952
B6	3.5	203	186	235850
US	3.7	363	273	372884
VX	3.2	55	31	36565
UA	2.9	704	183	196864
WN	5.3	650	591	1132745
FL	3.7	98	42	57563
OO	4.8	365	330	578300

TABLE S2. Comparison of the parameters in Complementary cumulative distribution functions (CCDFs) for model 1 with corresponding ones in the transfer function $q(l)$ in 2014.

airlines	codes	c_0	β_1	α_0	α	β_2
American Airlines	AA	7.77×10^2	40	2.03	0.43	35
American Eagle	MQ	3.74×10^3	50	2.84	0.49	40
Frontier Airlines	F9	5.37×10^6	90	3.59	0.59	90
Delta Airlines	DL	4.67×10^3	40	2.51	0.37	30
Hawaiian Airlines	HA	9.48×10^4	30	3.78	0.70	30
Alaska Airlines	AS	1.05×10^7	90	3.91	0.71	80

TABLE S3. Comparison of the parameters in CCDFs for model 2 with corresponding ones in the transfer function $q(l)$ in 2014.

airlines	codes	c_2	β_1	λ	r	α	β_2	m
JetBlue Airways	B6	1.18	8.73	81.97	0.50	0.52	8.70	15
Virgin America	VX	0.35	0.53	70.92	0.22	0.33	0.52	15
United Airlines	UA	1.99	8.47	97.07	0.63	0.41	8.45	8
US Airways	US	2.13	9.90	87.72	0.79	0.48	9.25	10
Southwest Airlines	WN	8.73	16.48	82.64	0.94	0.74	16.77	6
Atlantic Southeast Airlines	EV	0.79	6.62	79.37	0.33	0.53	6.86	21
AirTran Airways	FL	6.95	12.61	113.90	1.08	0.55	12.39	7
SkyWest Airlines	OO	1.50	11.83	83.33	0.57	0.75	11.95	24

DELAY PROBABILITY DISTRIBUTION FUNCTIONS FOR US AIRLINES FROM 1995 TO 2013

CCDFs for airlines in Group 1 (Fig. S1 — Fig. S10)

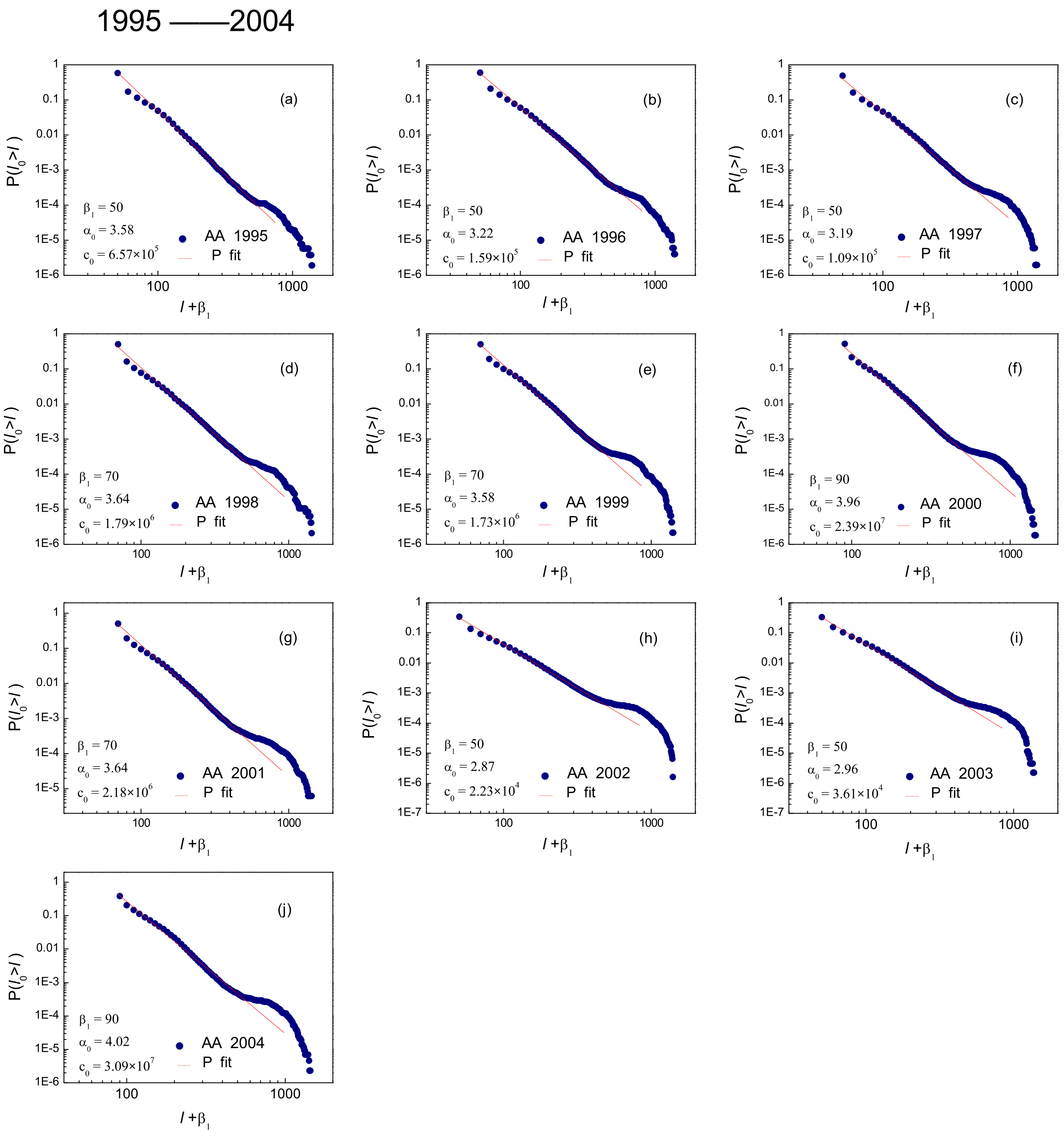

FIG. S1. Complementary cumulative distribution functions of departure delays l for airline AA from 1995 to 2004 ((a) - (j)). Red lines represent formula (8)($P = c_0(l+\beta_1)^{-\alpha_0}$) fitting CCDFs mined from real data. $\Delta l =$ *10min*.

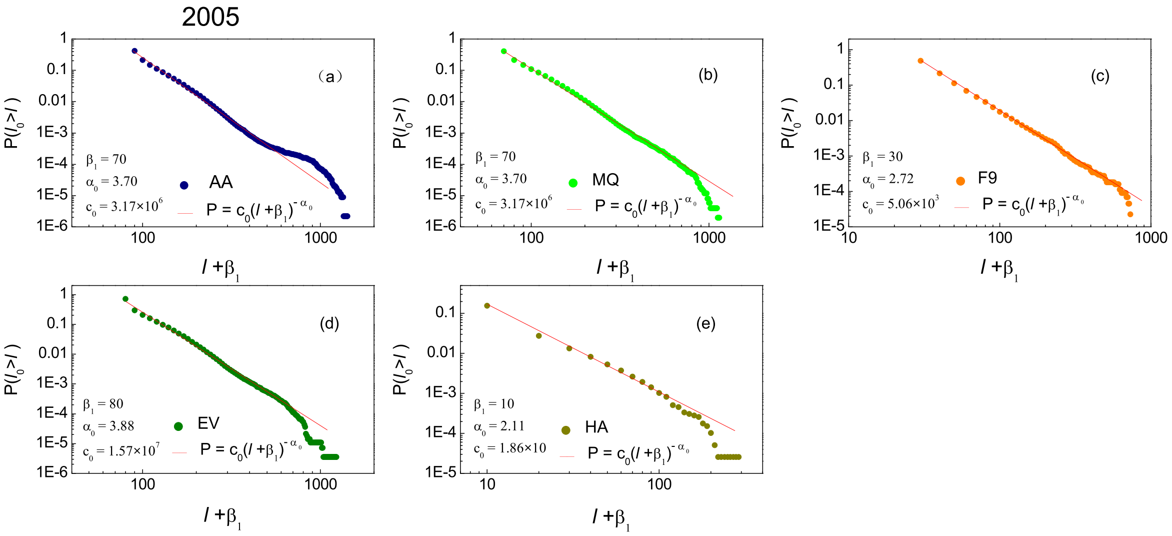

FIG. S2. Complementary cumulative distribution functions of departure delays l for airline AA(a), MQ(b), F9(c), EV(d) and HA(e) in 2005. $\Delta l = 10min$.

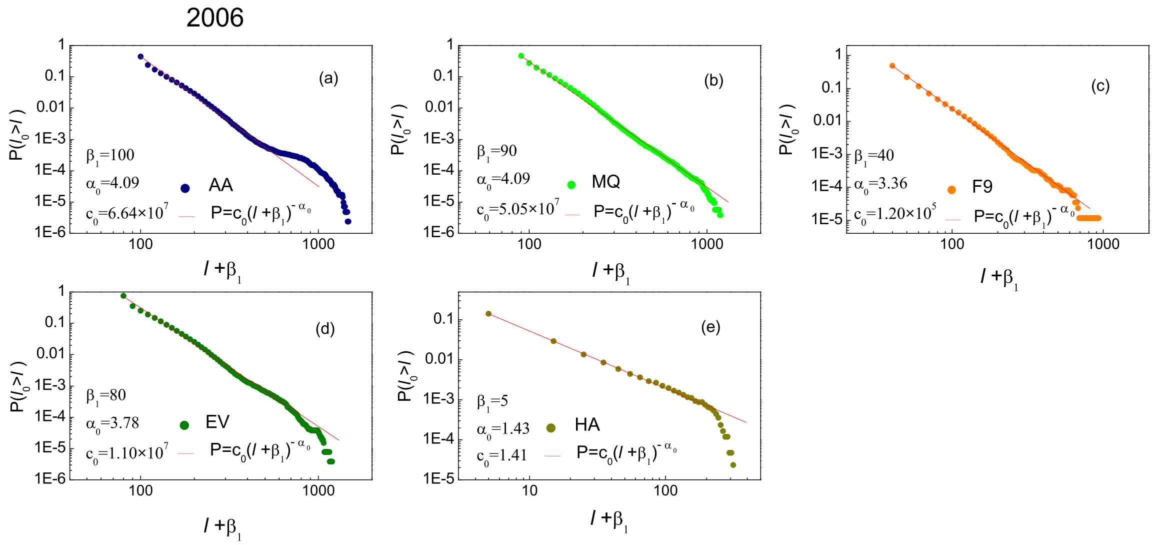

FIG. S3. Complementary cumulative distribution functions of departure delays l for airline AA(a), MQ(b), F9(c), EV(d) and HA(e) in 2006. $\Delta l =$ *10min*

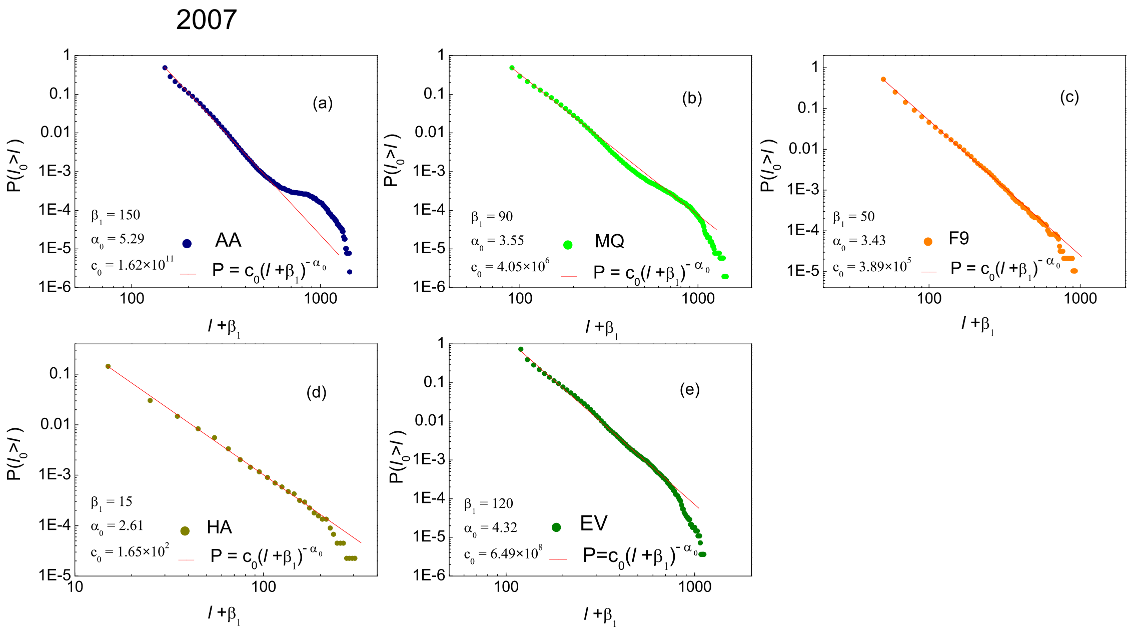

FIG. S4. Complementary cumulative distribution functions of departure delays l for airline AA(a), MQ(b), F9(c), HA(d) and EV(e) in 2007. $\Delta l = \mathit{10min}$

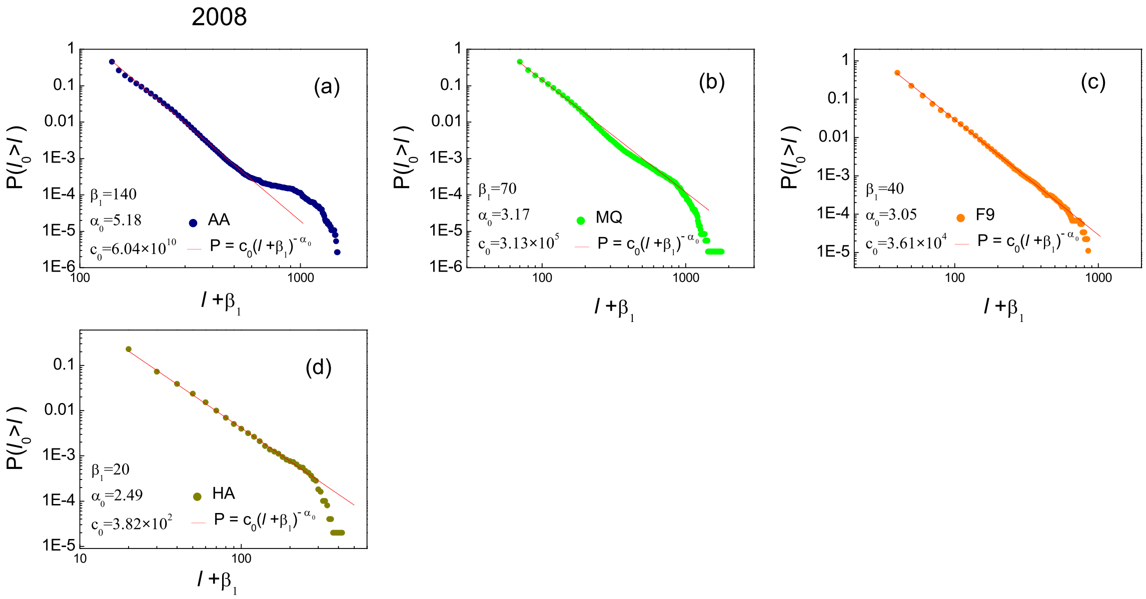

FIG. S5. Complementary cumulative distribution functions of departure delays l for airline AA(a), MQ(b), F9(c) and HA(d) in 2008. $\Delta l = \mathit{10min}$.

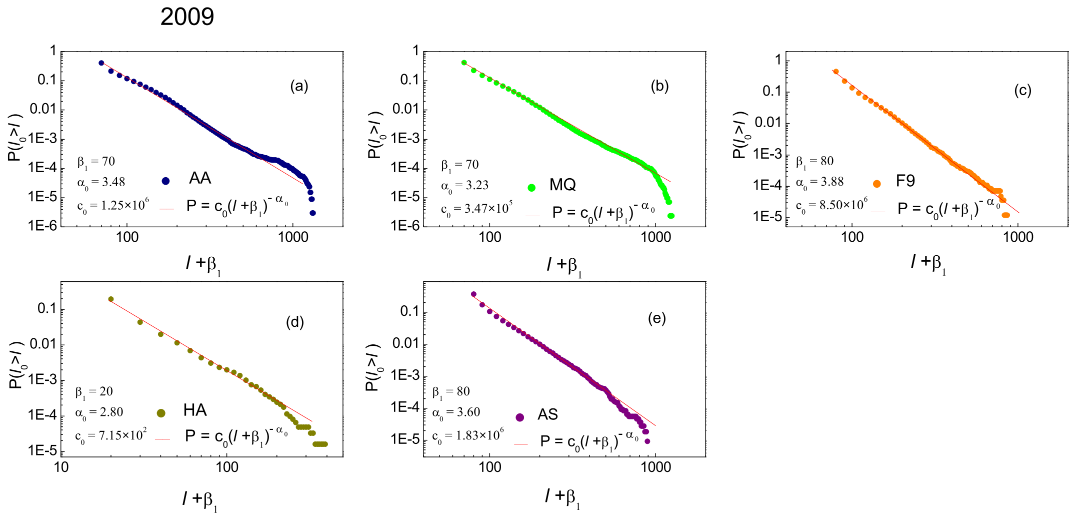

FIG. S6. Complementary cumulative distribution functions of departure delays l for airline AA(a), MQ(b), F9(c), HA(d) and AS(e) in 2009. $\Delta l =$ *10min*.

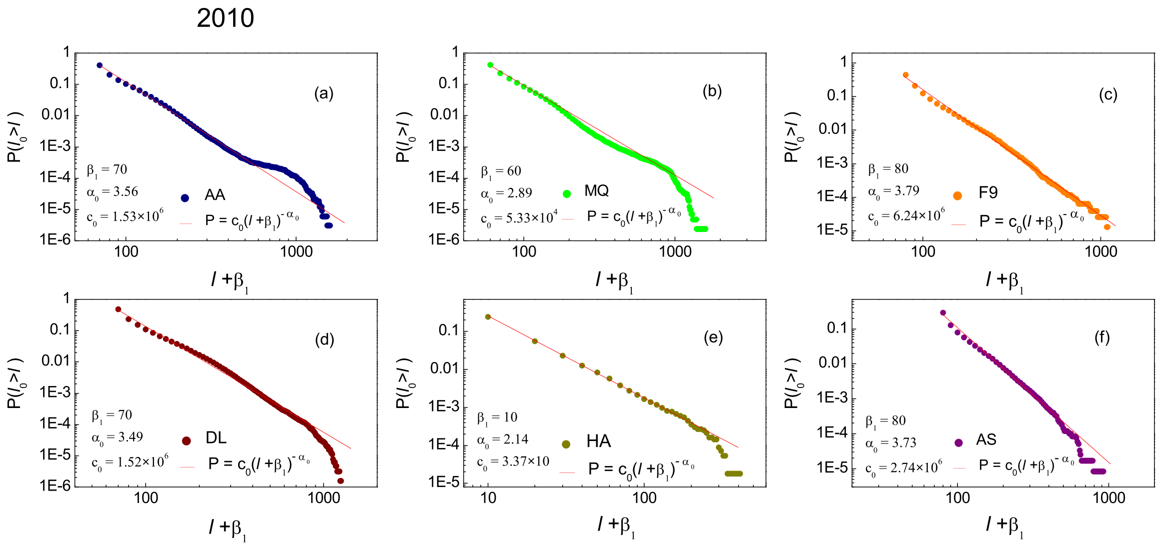

FIG. S7. Complementary cumulative distribution functions of departure delays l for airline AA(a), MQ(b), F9(c), DL(d), HA(e) and AS(f) in 2010. $\Delta l =$ *10min*.

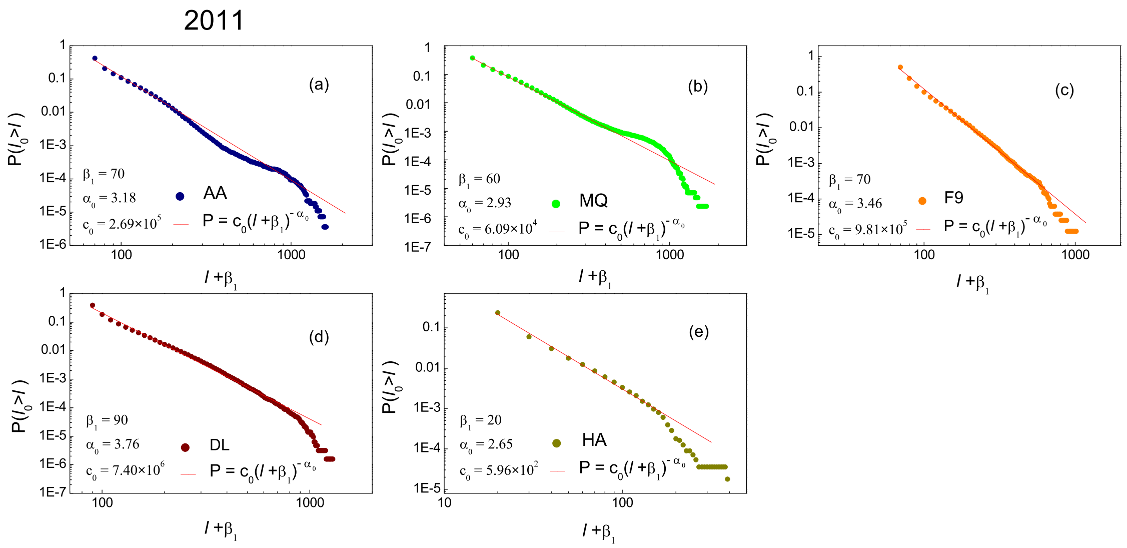

FIG. S8. Complementary cumulative distribution functions of departure delays l for airline AA(a), MQ(b), F9(c), DL(d) and HA(e) in 2011. $\Delta l =$ *10min*.

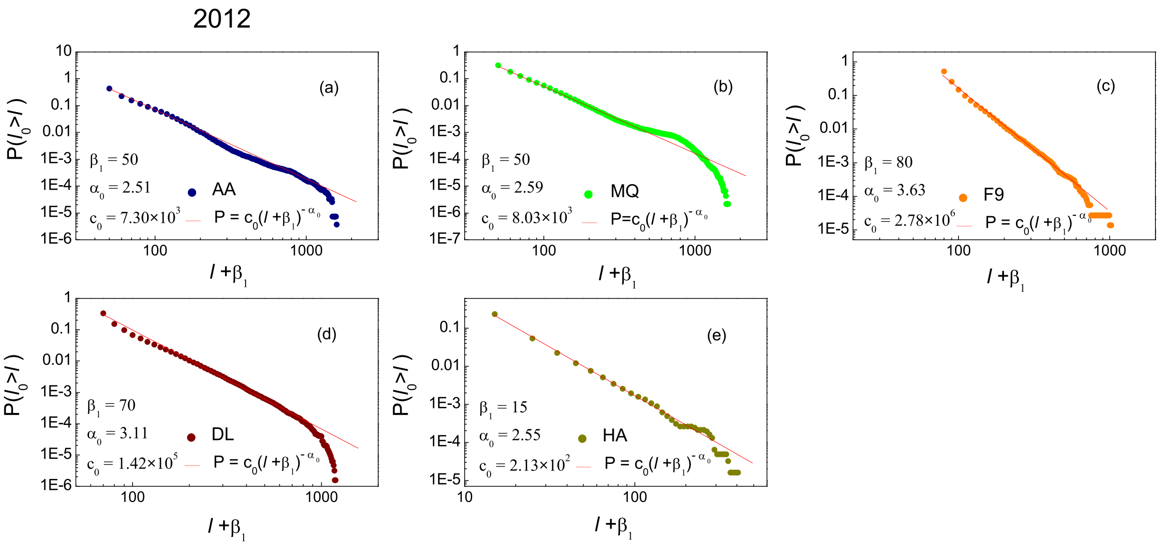

FIG. S9. Complementary cumulative distribution functions of departure delays l for airline AA(a), MQ(b), F9(c), DL(d) and HA(e) in 2012. $\Delta l =$ *10min*.

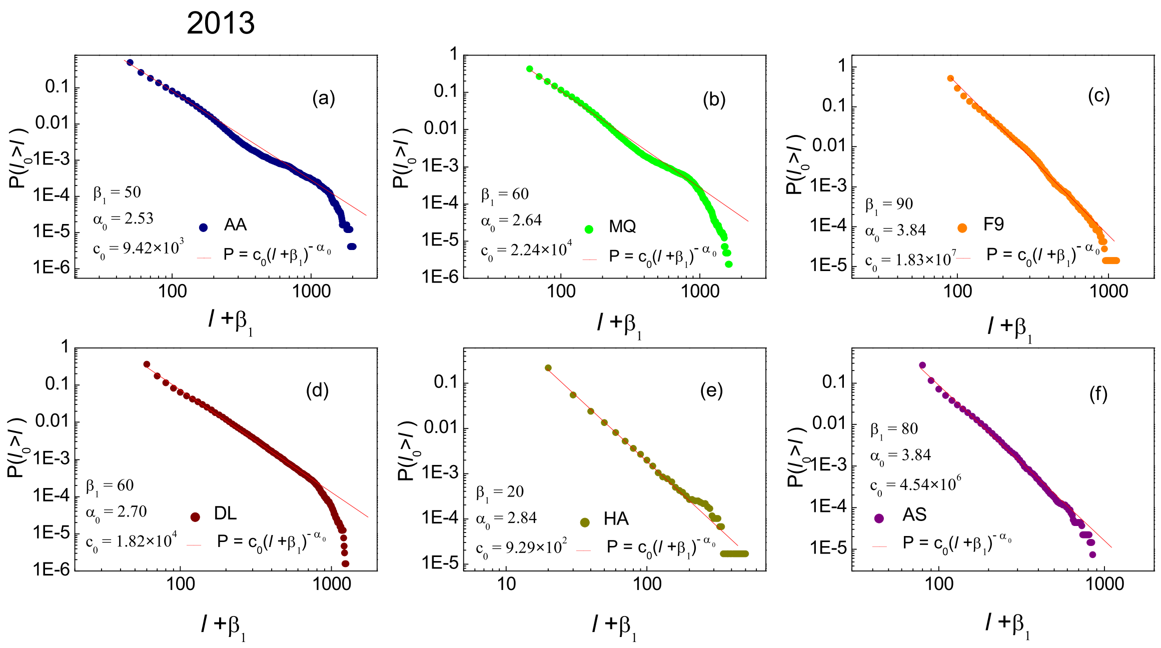

FIG. S10. Complementary cumulative distribution functions of departure delays l for airline AA(a), MQ(b), F9(c), DL(d), HA(e) and AS(f) in 2013. $\Delta l = 10min$.

CCDFs for airlines in Group 2 (Fig. S11 — Fig. S29)

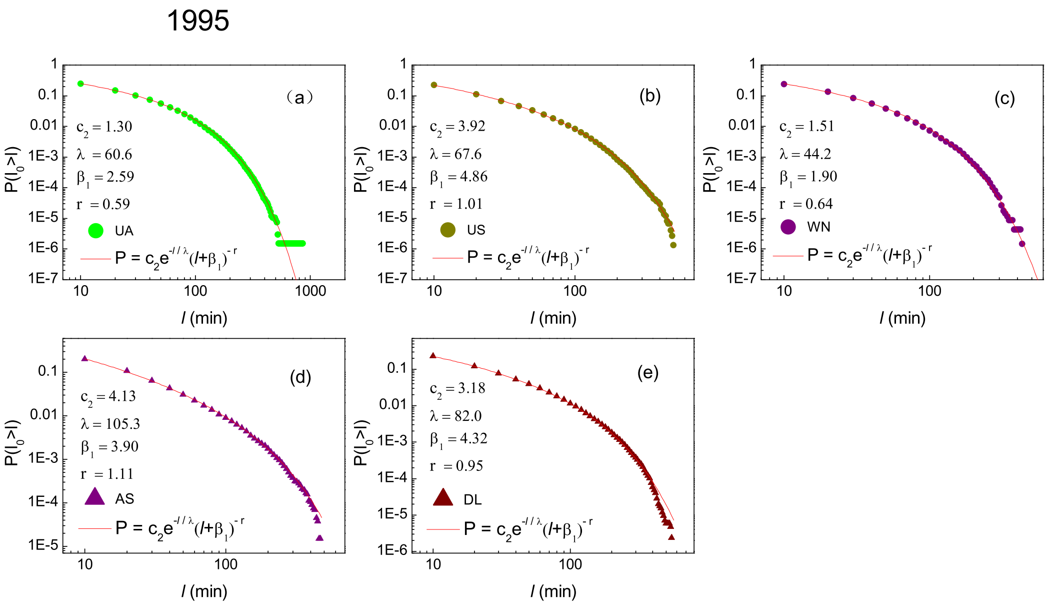

FIG. S11. Complementary cumulative distribution functions of departure delays l for airline UA(a), US(b), WN(c), AS(d) and DL(e) in 1995. $\Delta l = 10min$.

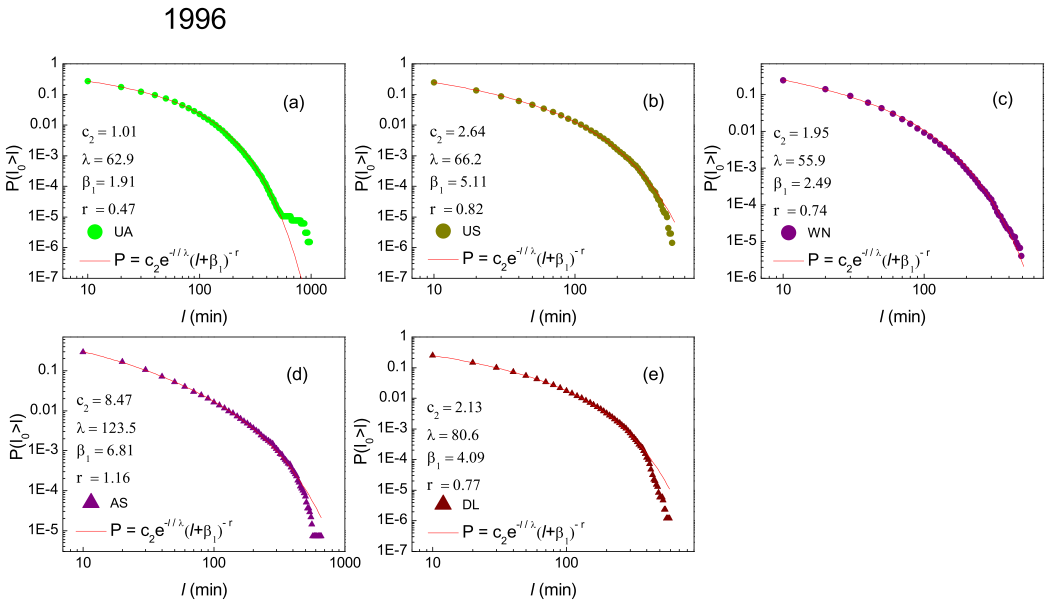

FIG. S12. Complementary cumulative distribution functions of departure delays l for airline UA(a), US(b), WN(c), AS(d) and DL(e) in 1996. $\Delta l = 10min$.

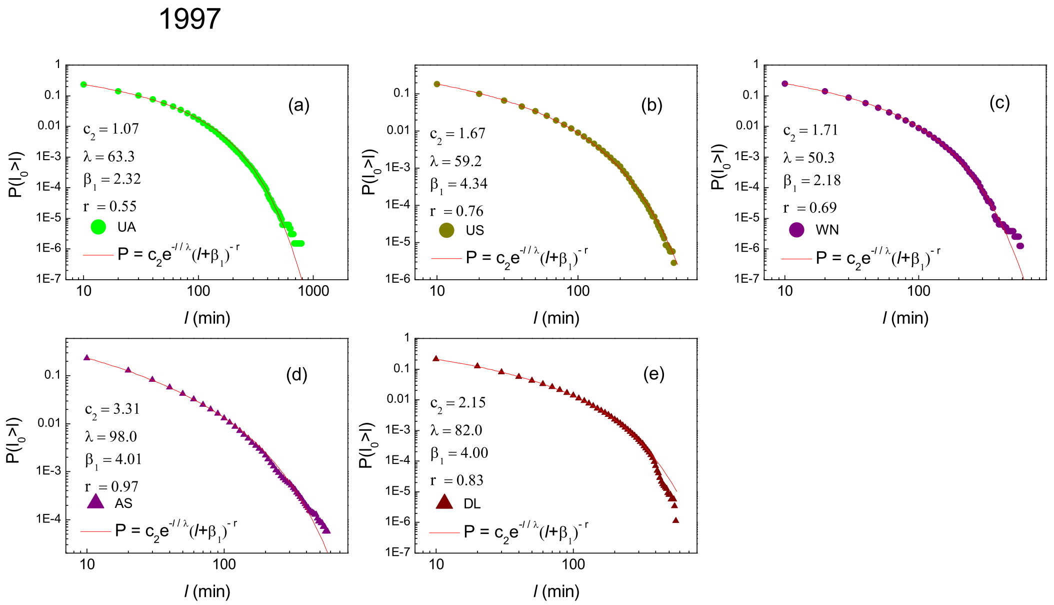

FIG. S13. Complementary cumulative distribution functions of departure delays l for airline UA(a), US(b), WN(c), AS(d) and DL(e) in 1997. $\Delta l = \mathit{10min}$.

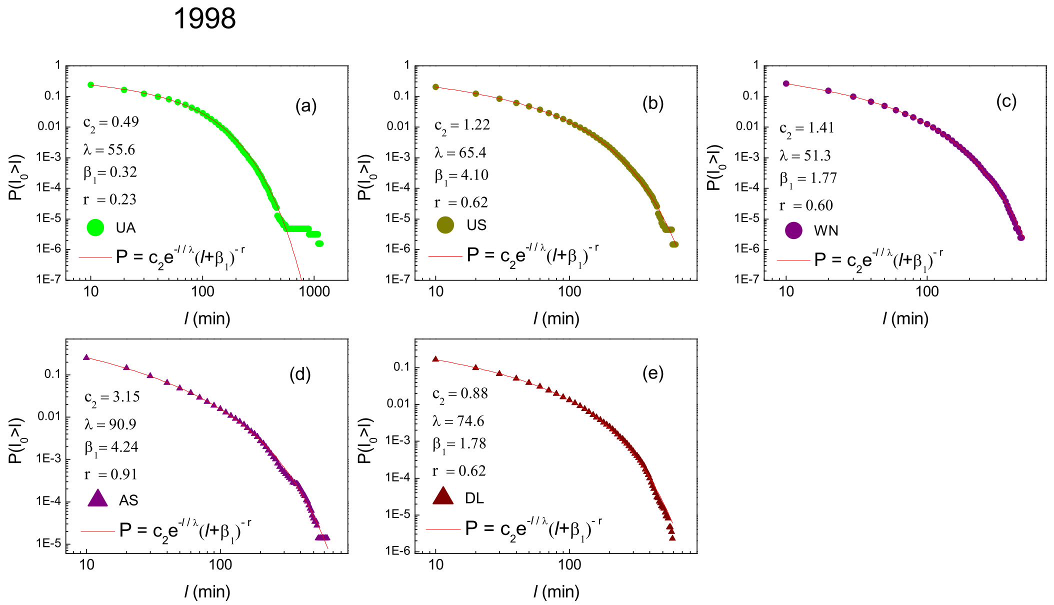

FIG. S14. Complementary cumulative distribution functions of departure delays l for airline UA(a), US(b), WN(c), AS(d) and DL(e) in 1998. $\Delta l = \mathit{10min}$.

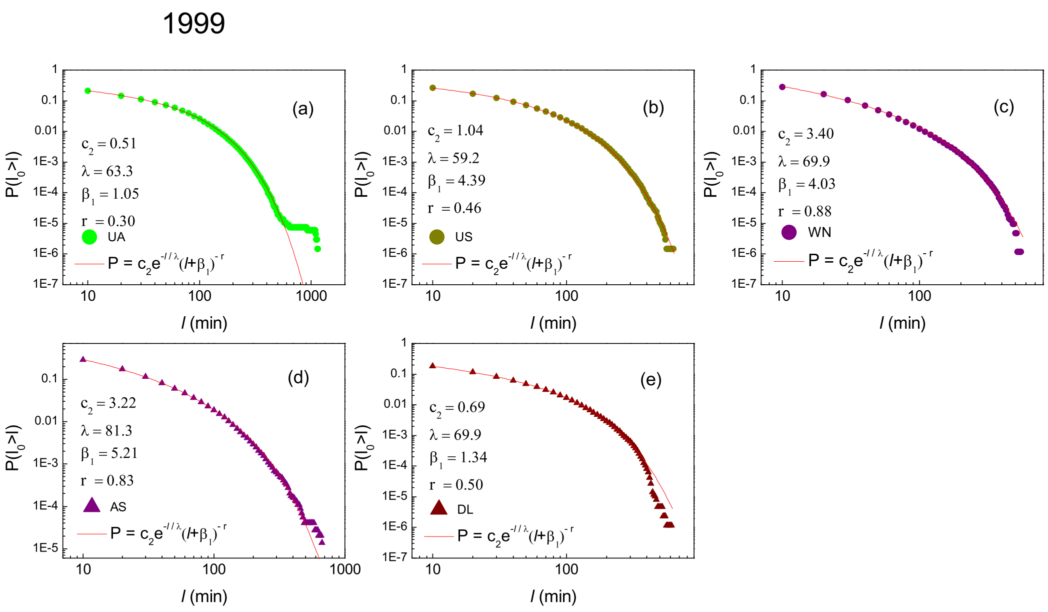

FIG. S15. Complementary cumulative distribution functions of departure delays l for airline UA(a), US(b), WN(c), AS(d) and DL(e) in 1999. Δl = *10min*.

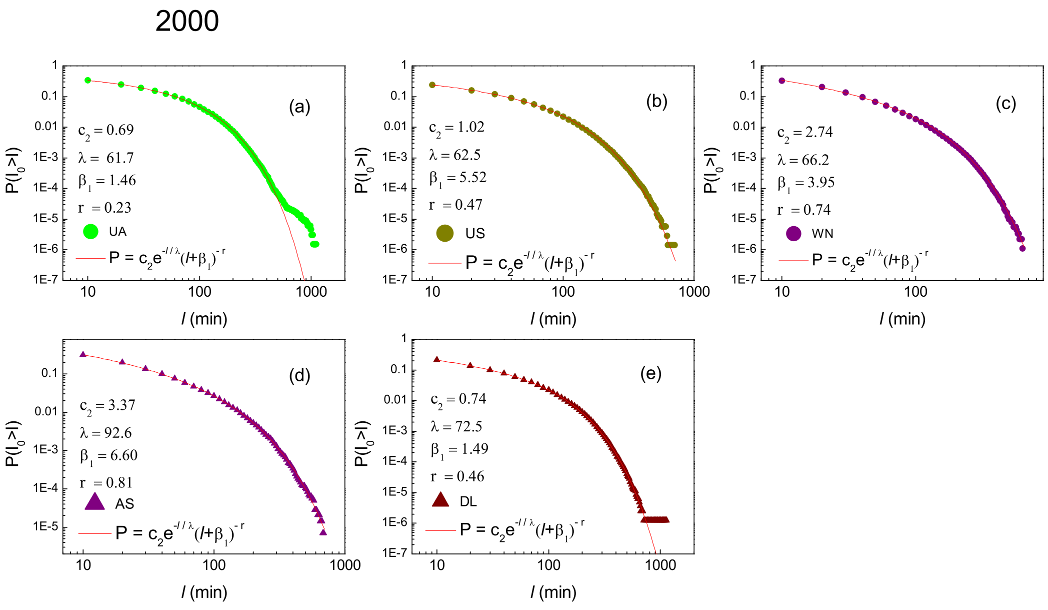

FIG. S16. Complementary cumulative distribution functions of departure delays l for airline UA(a), US(b), WN(c), AS(d) and DL(e) in 2000. Δl = *10min*.

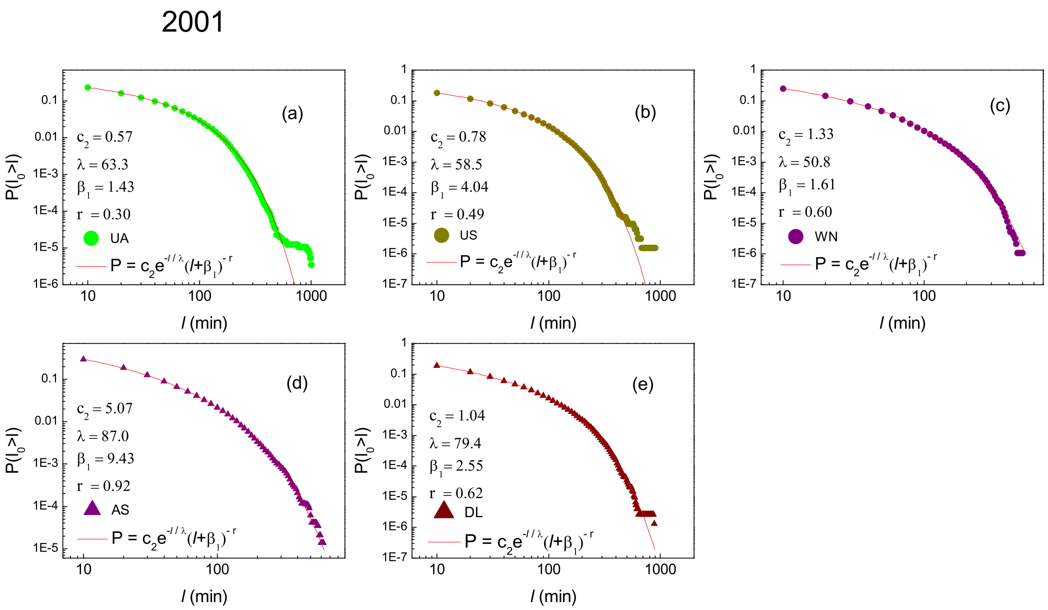

FIG. S17. Complementary cumulative distribution functions of departure delays l for airline UA(a), US(b), WN(c), AS(d) and DL(e) in 2001. $\Delta l = \mathit{10min}$.

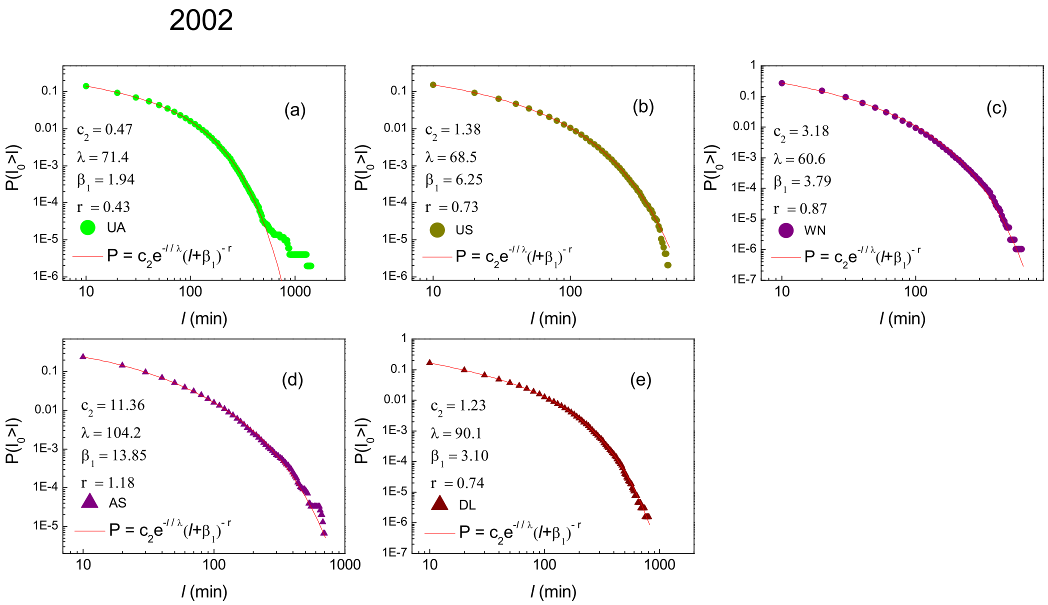

FIG. S18. Complementary cumulative distribution functions of departure delays l for airline UA(a), US(b), WN(c), AS(d) and DL(e) in 2002. $\Delta l = \mathit{10min}$.

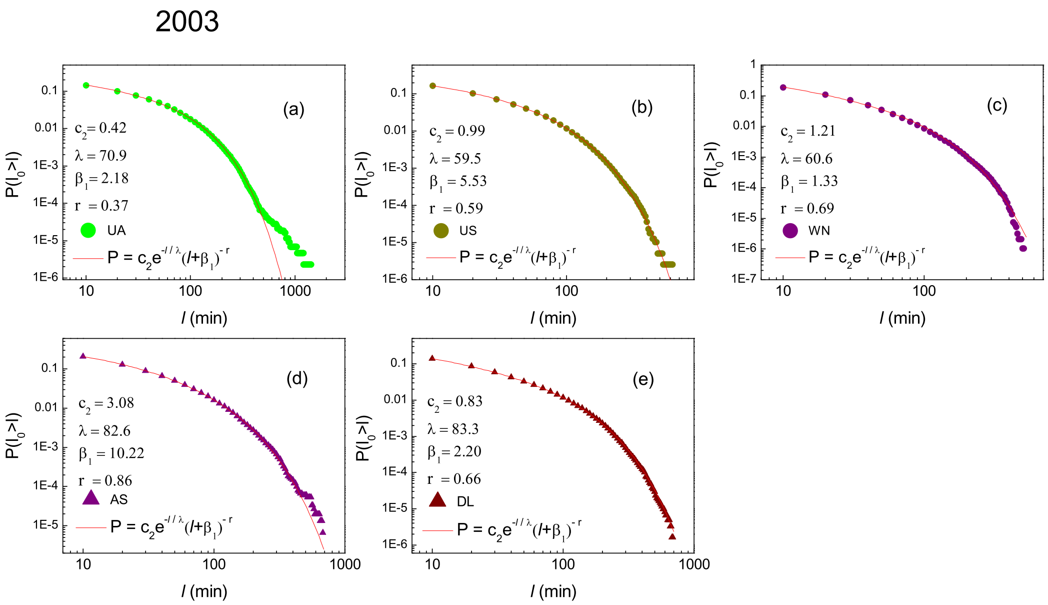

FIG. S19. Complementary cumulative distribution functions of departure delays l for airline UA(a), US(b), WN(c), AS(d) and DL(e) in 2003. $\Delta l = 10min$.

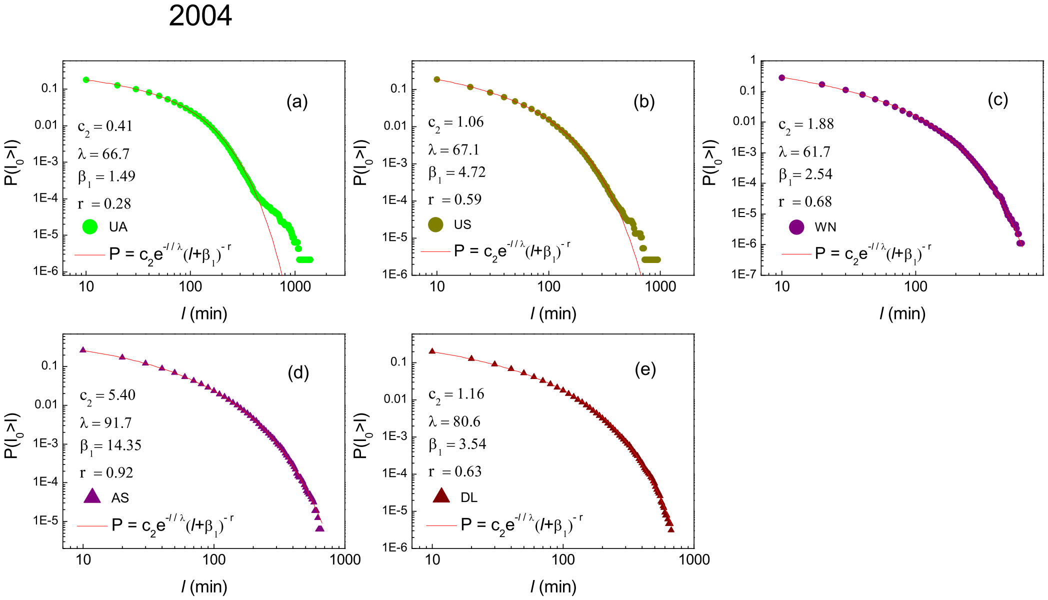

FIG. S20. Complementary cumulative distribution functions of departure delays l for airline UA(a), US(b), WN(c), AS(d) and DL(e) in 2004. $\Delta l = 10min$.

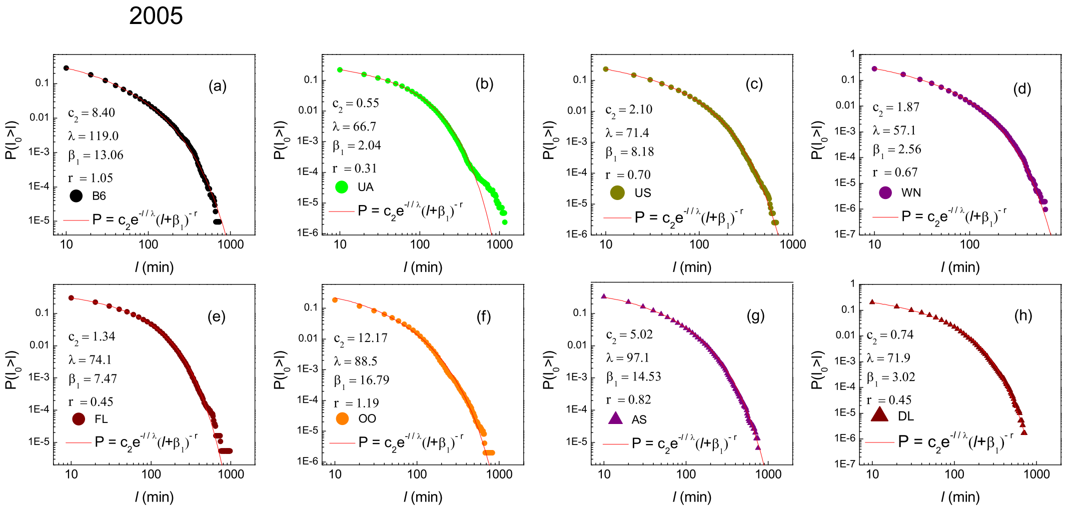

FIG. S21. Complementary cumulative distribution functions of departure delays l for airline AA(a), MQ(b), F9(c), DL(d) and HA(e) in 2012. Δl = *10min*.

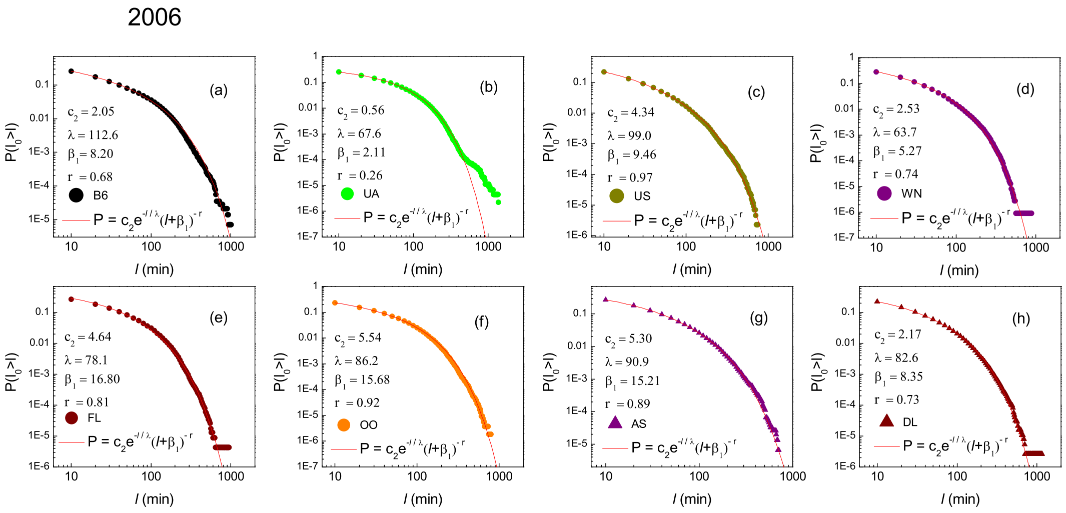

FIG. S22. Complementary cumulative distribution functions of departure delays l for airline B6(a),UA(b), US(c), WN(d), FL(e), OO(f), AS(g) and DL(h) in 2006. Δl = *10min*.

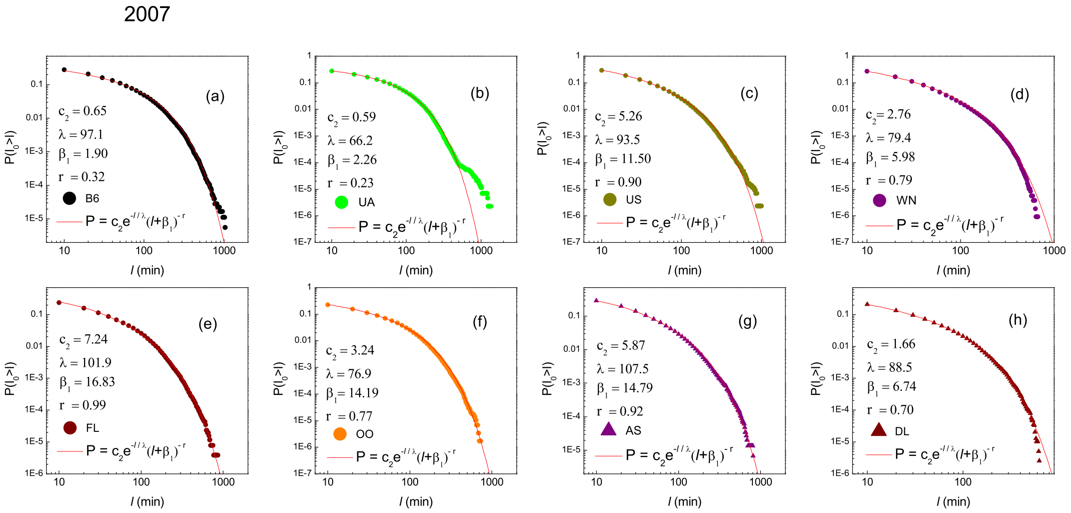

FIG. S23. Complementary cumulative distribution functions of departure delays l for airline B6(a),UA(b), US(c), WN(d), FL(e), OO(f), AS(g) and DL(h) in 2007. $\Delta l = 10min$.

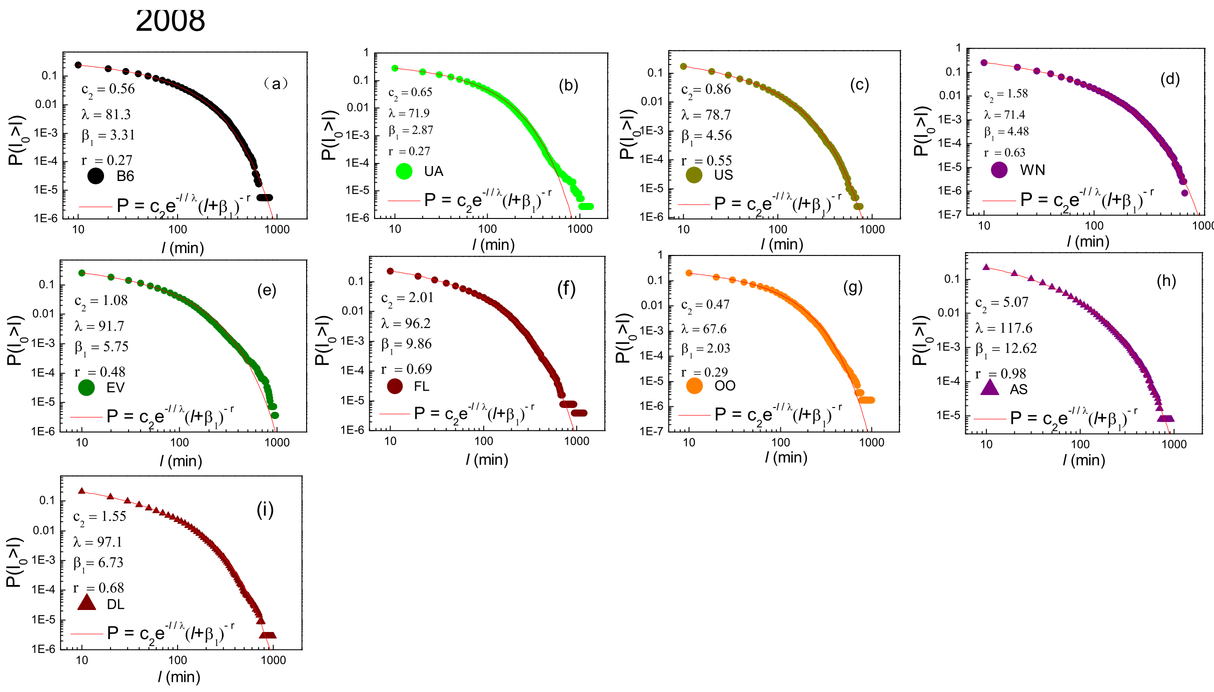

FIG. S24. Complementary cumulative distribution functions of departure delays l for airline B6(a),UA(b), US(c), WN(d), EV(e), FL(f), OO(g), AS(h) and DL(i) in 2008. $\Delta l = 10min$.

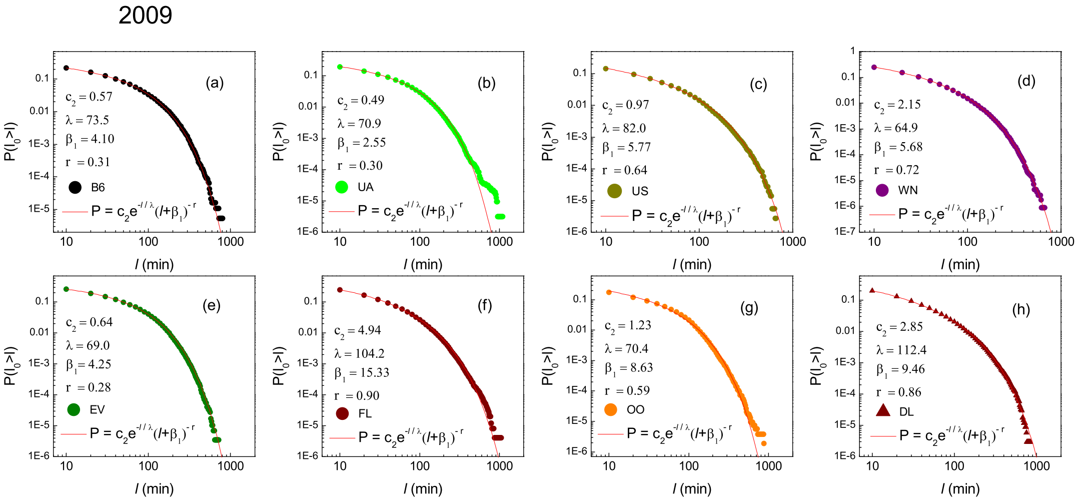

FIG. S25. Complementary cumulative distribution functions of departure delays l for airline B6(a),UA(b), US(c), WN(d), EV(e), FL(f), OO(g) and DL(h) in 2009. $\Delta l = \mathit{10min}$.

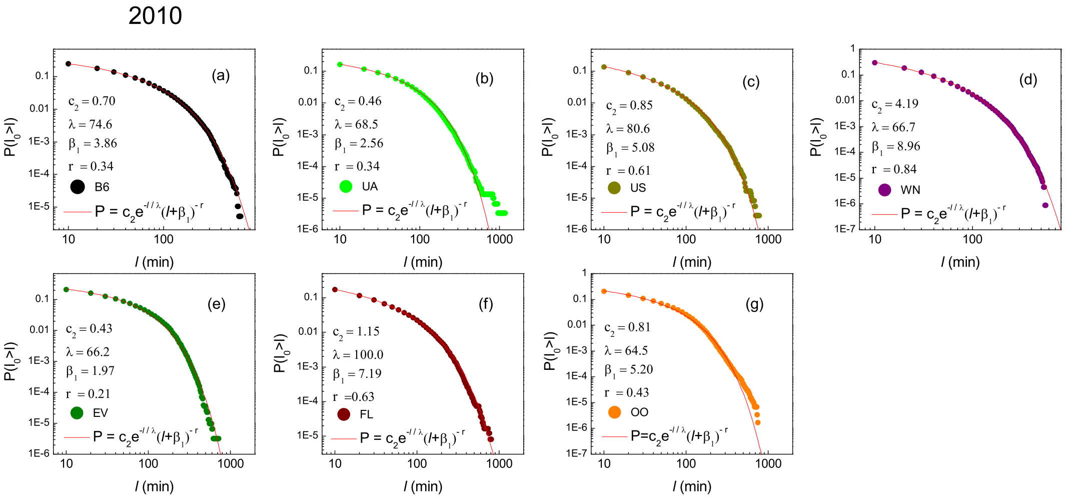

FIG. S26. Complementary cumulative distribution functions of departure delays l for airline B6(a),UA(b), US(c), WN(d), EV(e), FL(f) and OO(g) in 2010. $\Delta l = \mathit{10min}$.

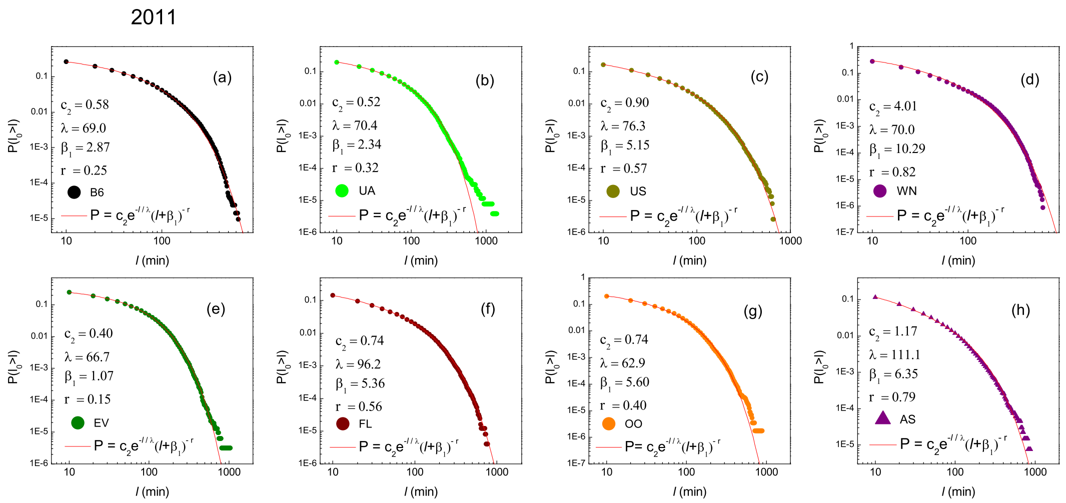

FIG. S27. Complementary cumulative distribution functions of departure delays l for airline B6(a),UA(b), US(c), WN(d), EV(e), FL(f), OO(g) and AS(h) in 2011. $\Delta l =$ *10min*.

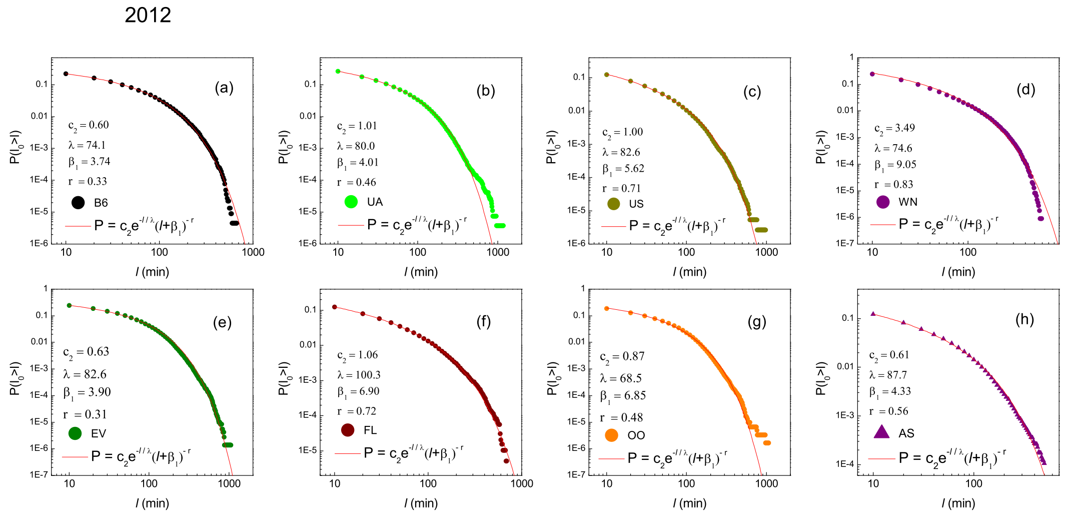

FIG. S28. Complementary cumulative distribution functions of departure delays l for airline B6(a),UA(b), US(c), WN(d), EV(e), FL(f), OO(g) and AS(h) in 2012. $\Delta l =$ *10min*.

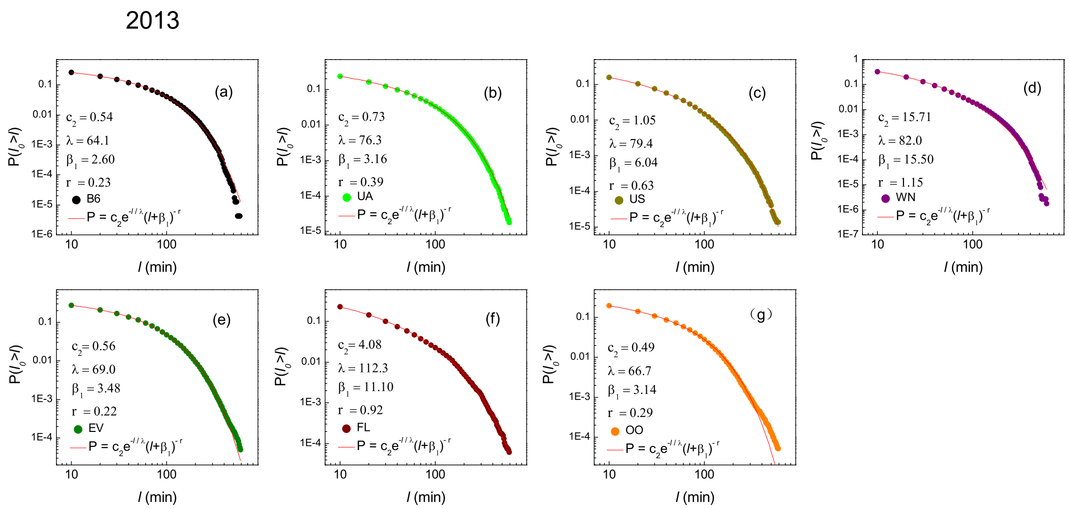

FIG. S29. Complementary cumulative distribution functions of departure delays l for airline B6(a),UA(b), US(c), WN(d), EV(e), FL(f) and OO(g) in 2013. $\Delta l = \mathit{10min}$.

Cumulated transfer function $F(l)$ in Group 1 (Fig. S30 — Fig. S39)

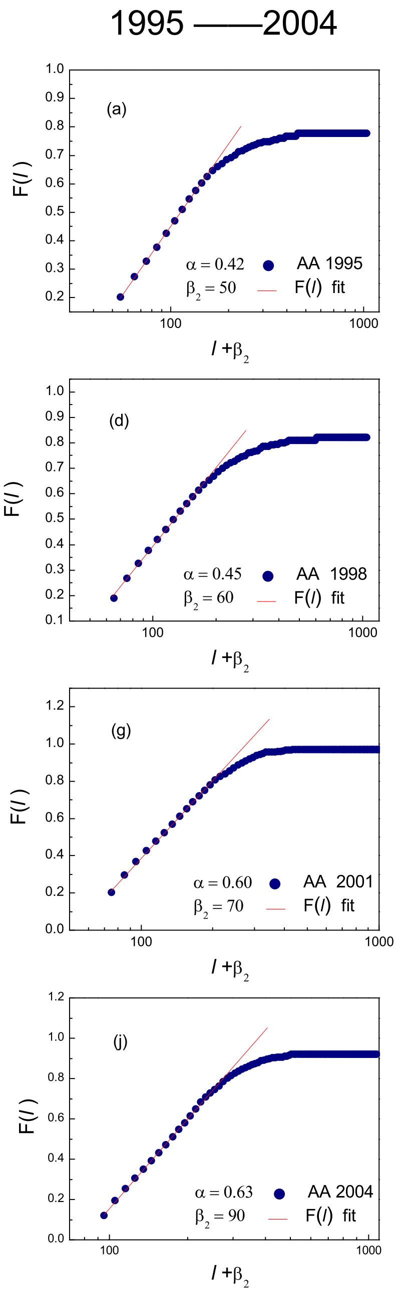

FIG. S30. Complementary cumulative distribution functions of departure delays l for airline AA(a), MQ(b), F9(c), EV(d) and HA(e) from 1995 to 2004. $\Delta l =$ *10min*.

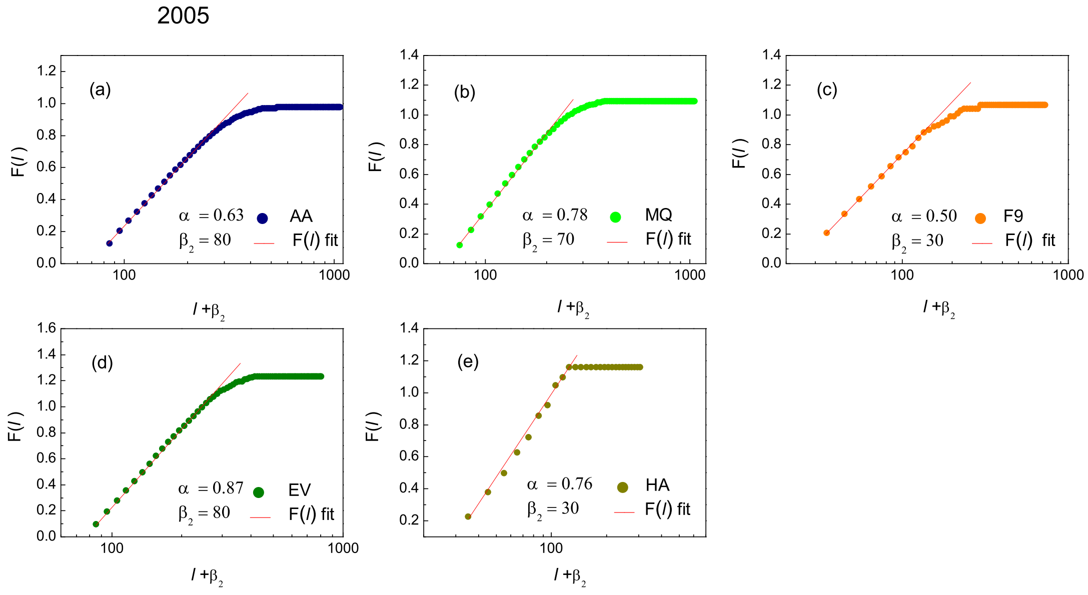

FIG. S31. Complementary cumulative distribution functions of departure delays l for airline AA(a), MQ(b), F9(c), EV(d) and HA(e) in 2005. Δl = *10min*.

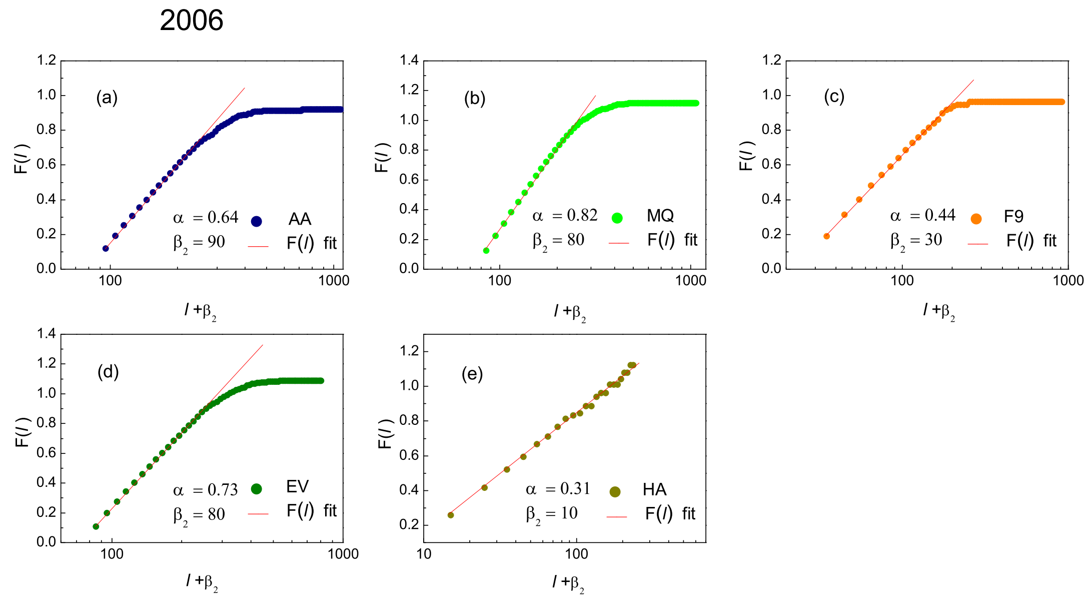

FIG. S32. Complementary cumulative distribution functions of departure delays l for airline AA(a), MQ(b), F9(c), EV(d) and HA(e) in 2006. Δl = *10min*.

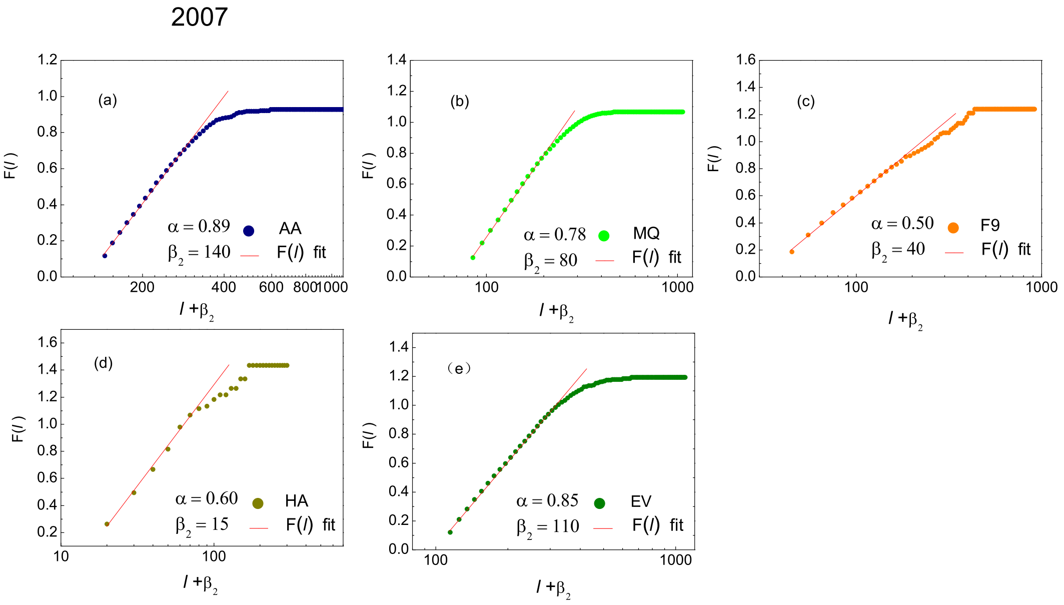

FIG. S33. Complementary cumulative distribution functions of departure delays l for airline AA(a), MQ(b), F9(c), HA(d) and EV(e) in 2007. $\Delta l = \mathit{10min}$.

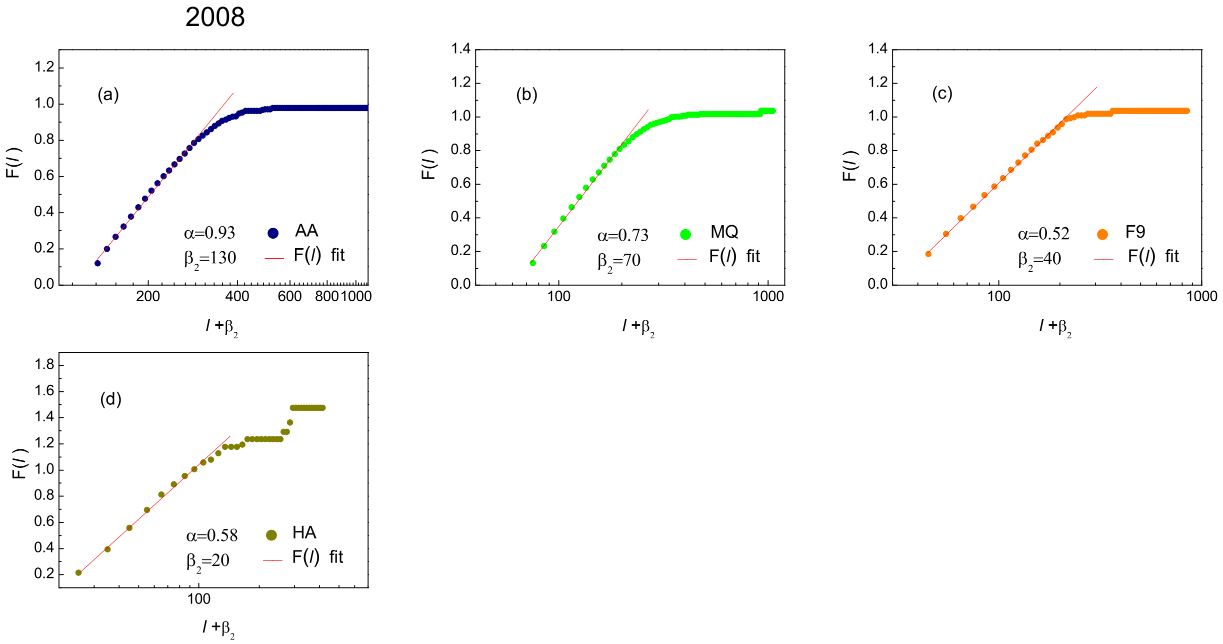

FIG. S34. Complementary cumulative distribution functions of departure delays l for airline AA(a), MQ(b), F9(c) and HA(d) in 2008. $\Delta l = \mathit{10min}$.

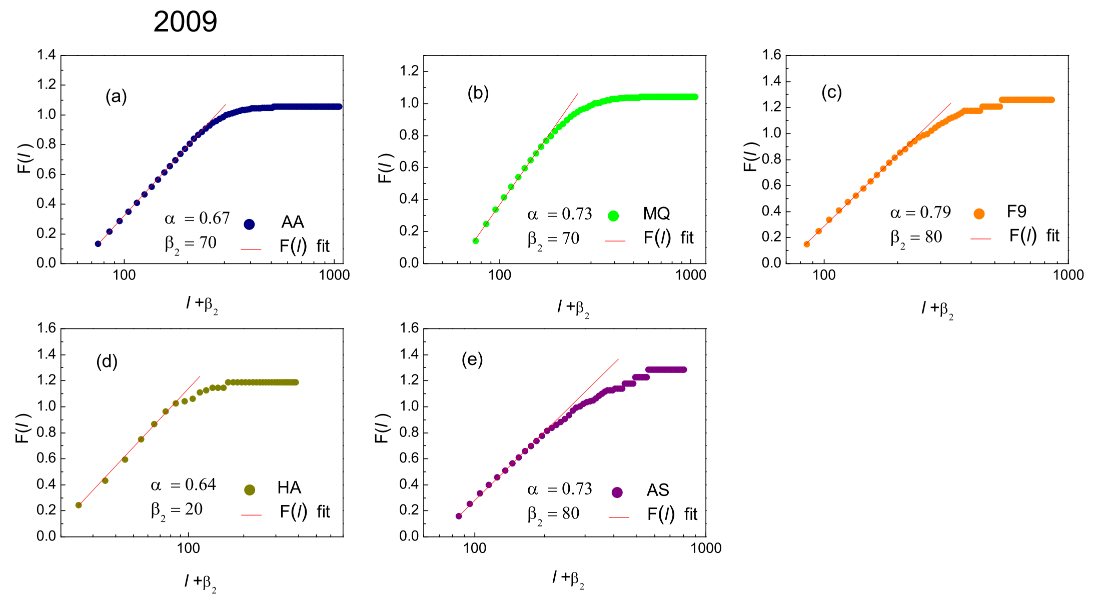

FIG. S35. Complementary cumulative distribution functions of departure delays l for airline AA(a), MQ(b), F9(c), HA(d) and AS(e) in 2009. $\Delta l =$ *10min*.

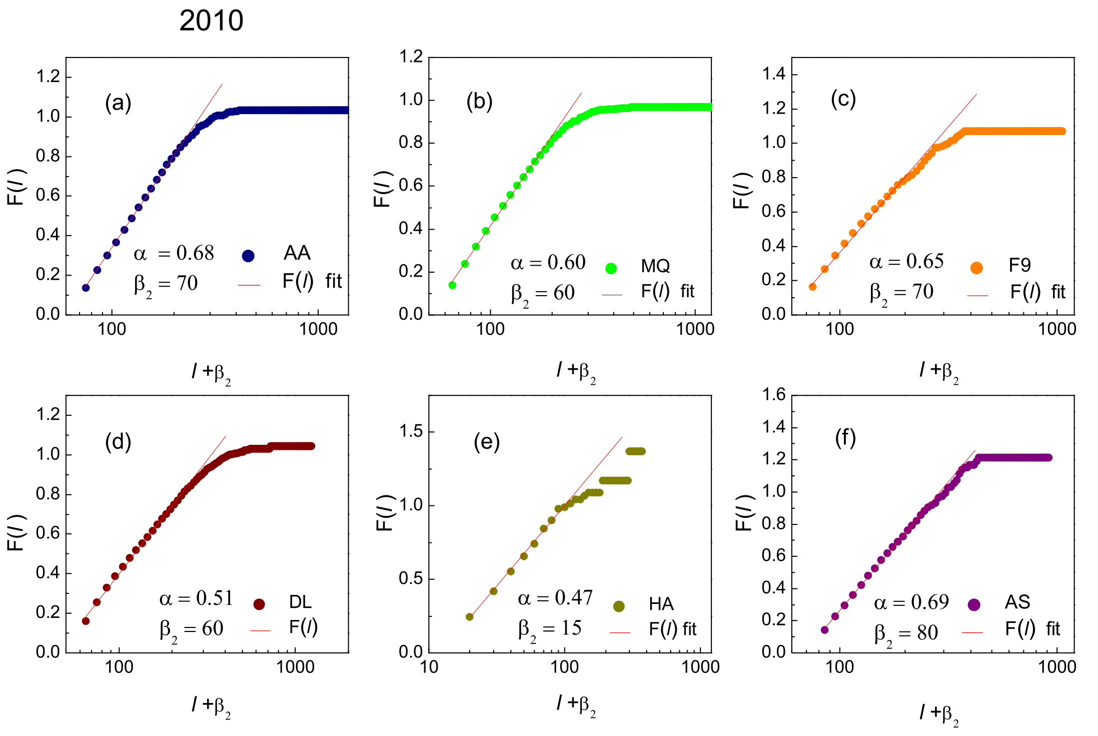

FIG. S36. Complementary cumulative distribution functions of departure delays l for airline AA(a), MQ(b), F9(c), DL(d), HA(e) and AS(f) in 2010. $\Delta l =$ *10min*.

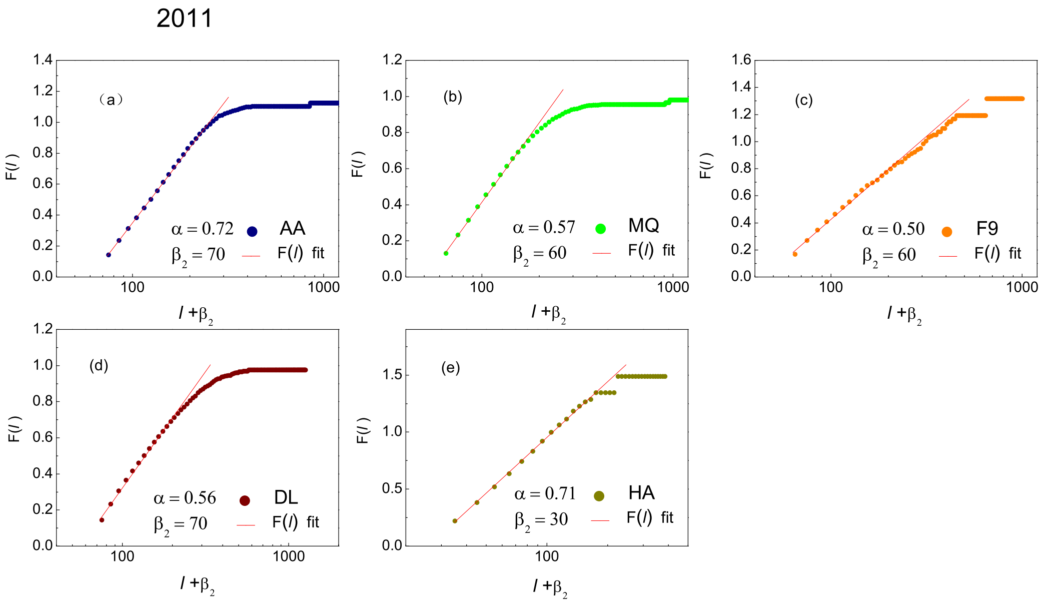

FIG. S37. Complementary cumulative distribution functions of departure delays l for airline AA(a), MQ(b), F9(c), DL(d) and HA(e) in 2011. $\Delta l = \mathit{10min}$.

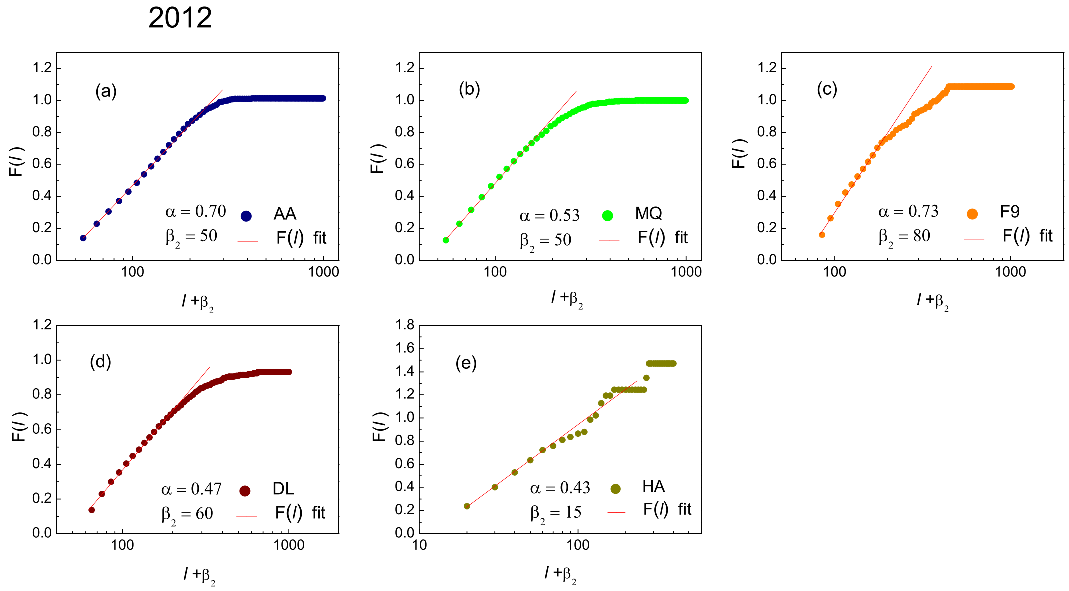

FIG. S38. Complementary cumulative distribution functions of departure delays l for airline AA(a), MQ(b), F9(c), DL(d) and HA(e) in 2012. $\Delta l = \mathit{10min}$.

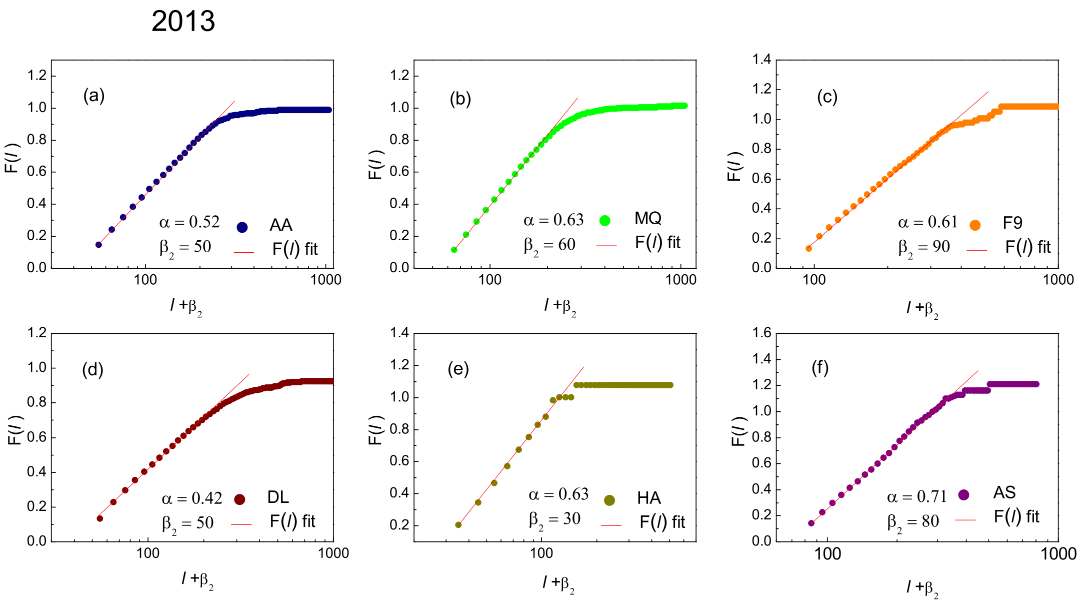

FIG. S39. Complementary cumulative distribution functions of departure delays l for airline AA(a), MQ(b), F9(c), DL(d), HA(e) and AS(f) in 2013. $\Delta l = \mathit{10min}$.

Cumulated transfer function $F(l)$ in Group 2(Fig. S40 — Fig. S58)

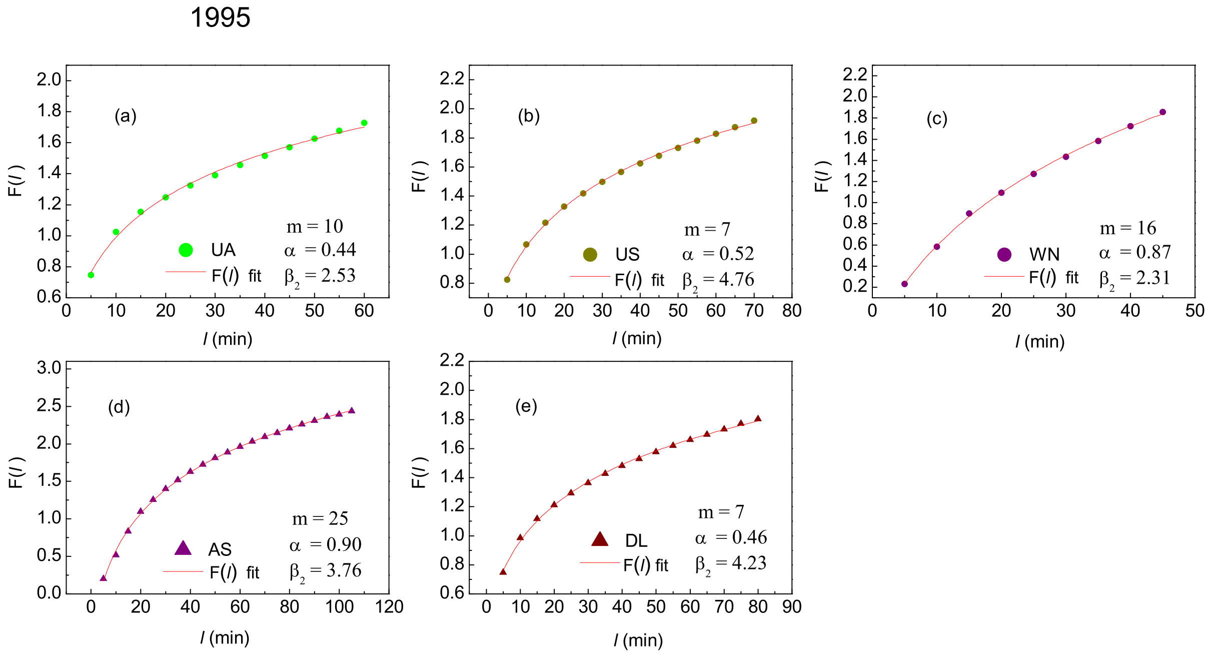

FIG. S40. $F(l) = \alpha ln(1 + \frac{l}{\beta_2})$(red lines) fitting cumulated transfer function mined from primary data of airline UA(a), US(b), WN(c), AS(d) and DL(e) in 1995. Δl = *5min*.

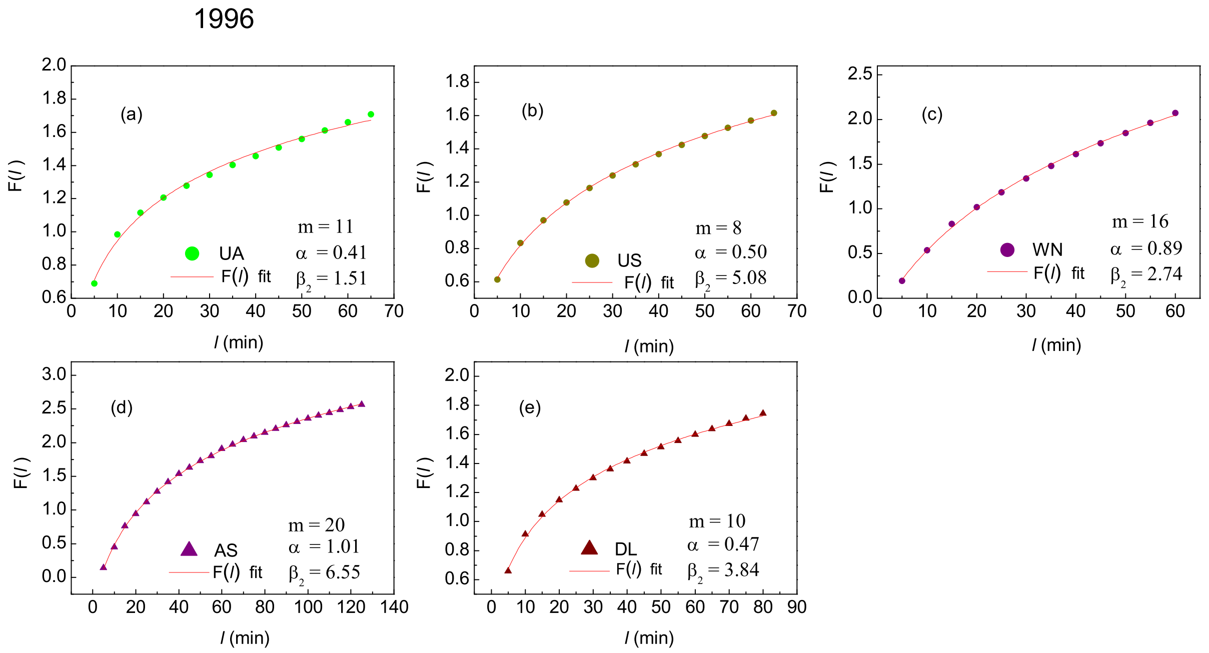

FIG. S41. $F(l) = \alpha ln(1 + \frac{l}{\beta_2})$(red lines) fitting cumulated transfer function mined from primary data of airline UA(a), US(b), WN(c), AS(d) and DL(e) in 1996. Δl = *5min*.

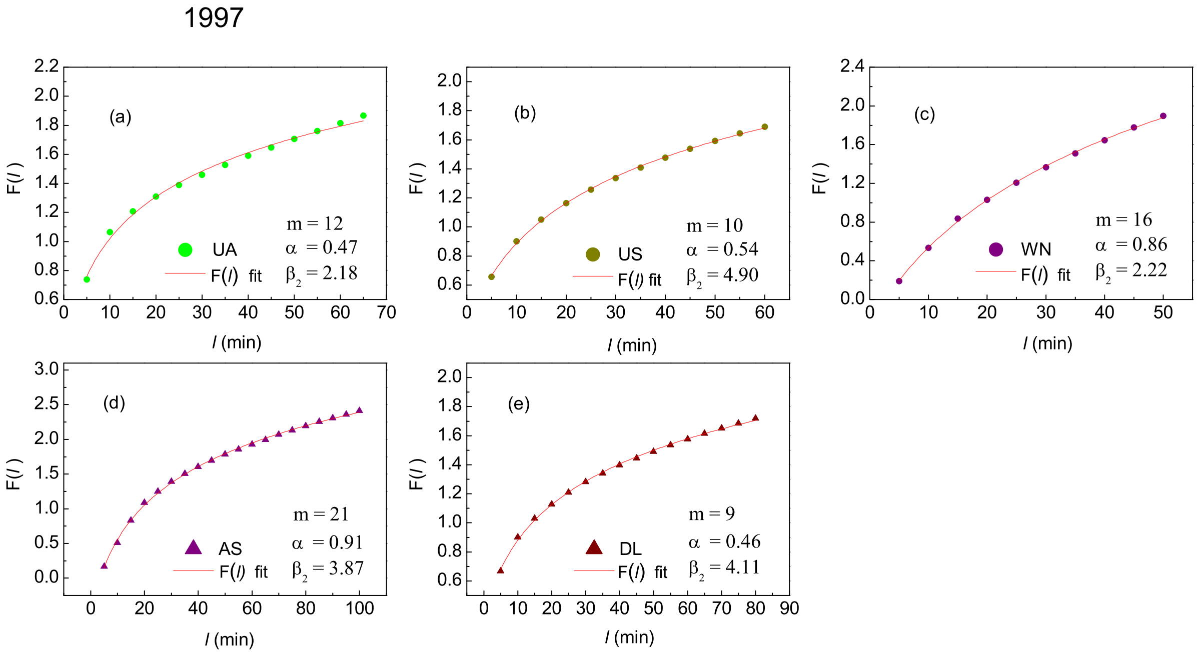

FIG. S42. $F(l) = \alpha ln(1 + \frac{l}{\beta_2})$(red lines) fitting cumulated transfer function mined from primary data of airline UA(a), US(b), WN(c), AS(d) and DL(e) in 1997. $\Delta l = 5min$.

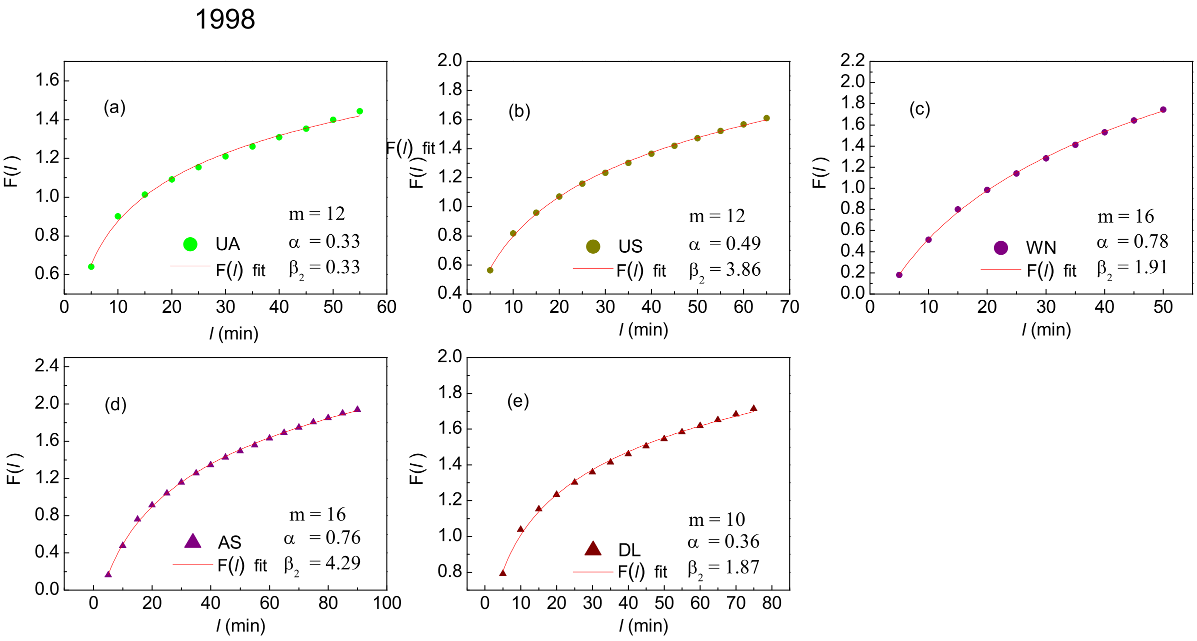

FIG. S43. $F(l) = \alpha ln(1 + \frac{l}{\beta_2})$(red lines) fitting cumulated transfer function mined from primary data of airline UA(a), US(b), WN(c), AS(d) and DL(e) in 1998. $\Delta l = 5min$.

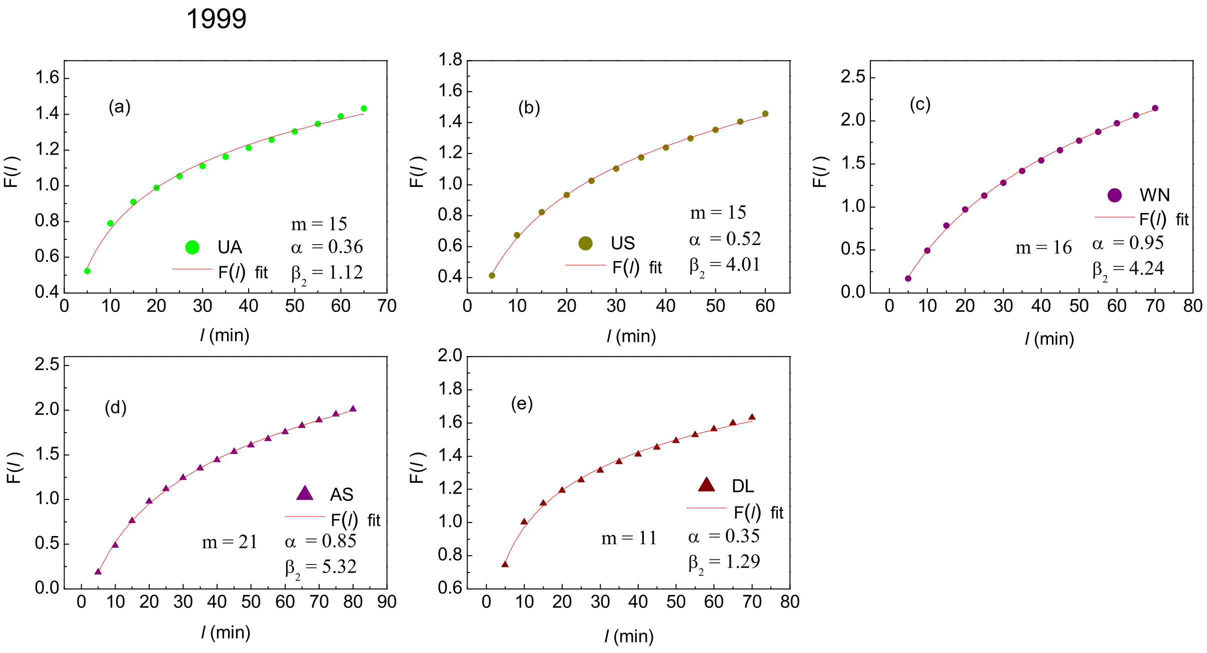

FIG. S44. $F(l) = \alpha ln(1 + \frac{l}{\beta_2})$(red lines) fitting cumulated transfer function mined from primary data of airline UA(a), US(b), WN(c), AS(d) and DL(e) in 1999. $\Delta l = 5min$.

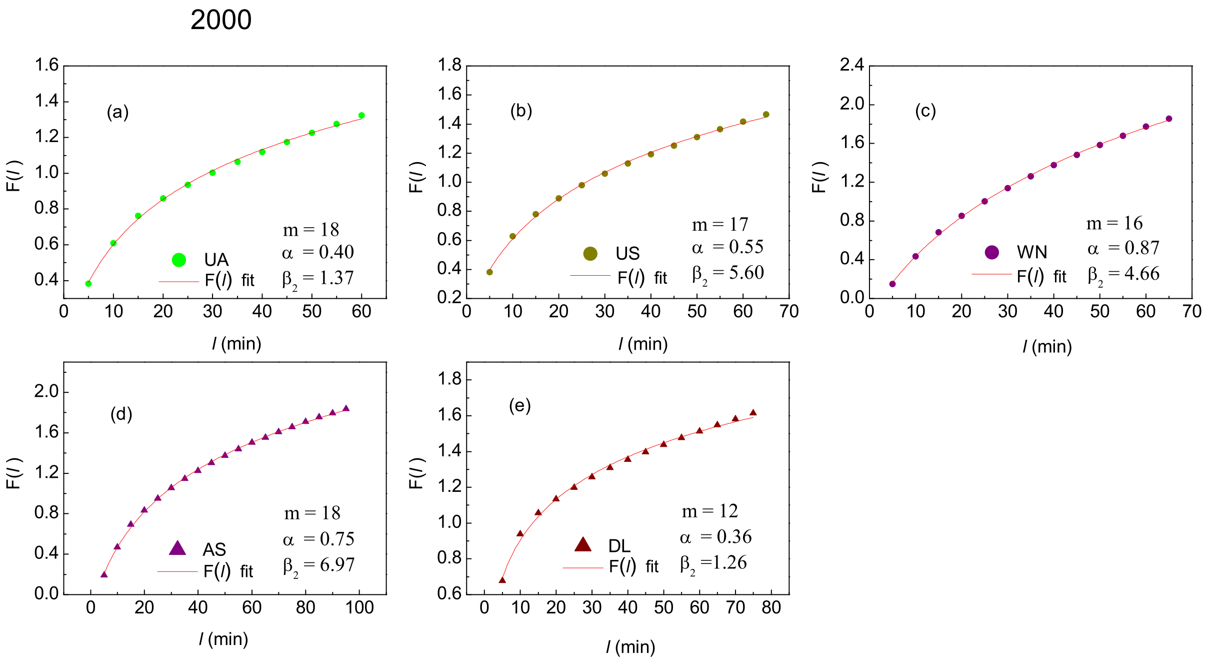

FIG. S45. $F(l) = \alpha ln(1 + \frac{l}{\beta_2})$(red lines) fitting cumulated transfer function mined from primary data of airline UA(a), US(b), WN(c), AS(d) and DL(e) in 2000. $\Delta l = 5min$.

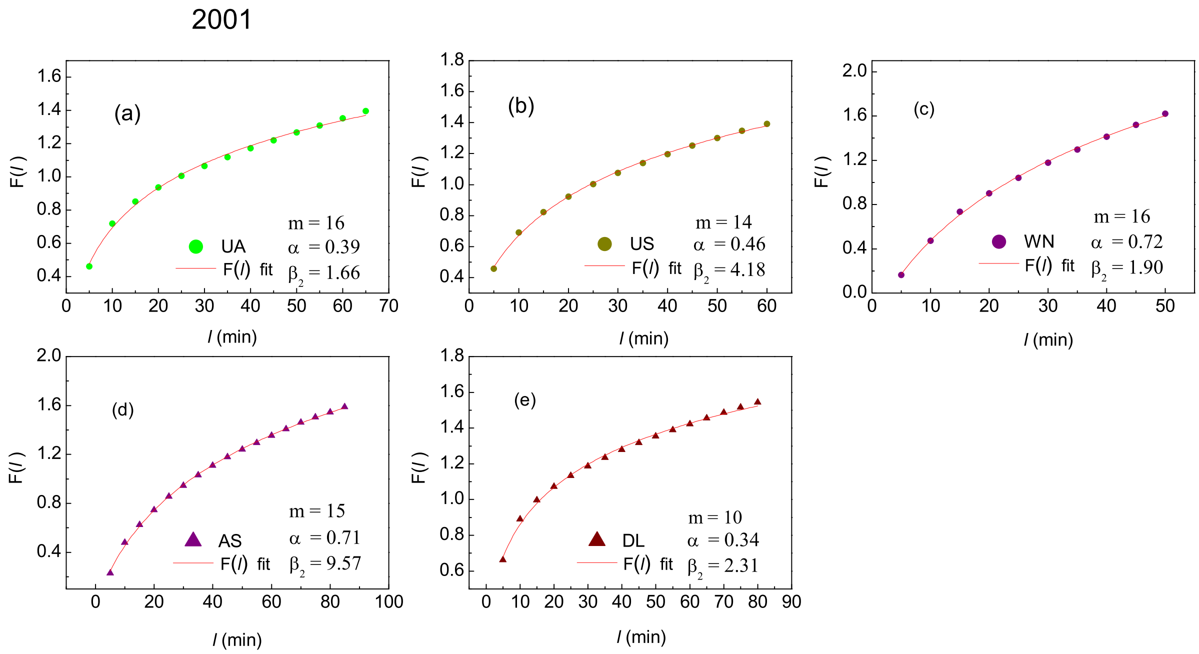

FIG. S46. $F(l) = \alpha ln(1 + \frac{l}{\beta_2})$(red lines) fitting cumulated transfer function mined from primary data of airline UA(a), US(b), WN(c), AS(d) and DL(e) in 2001. $\Delta l =$ *5min*.

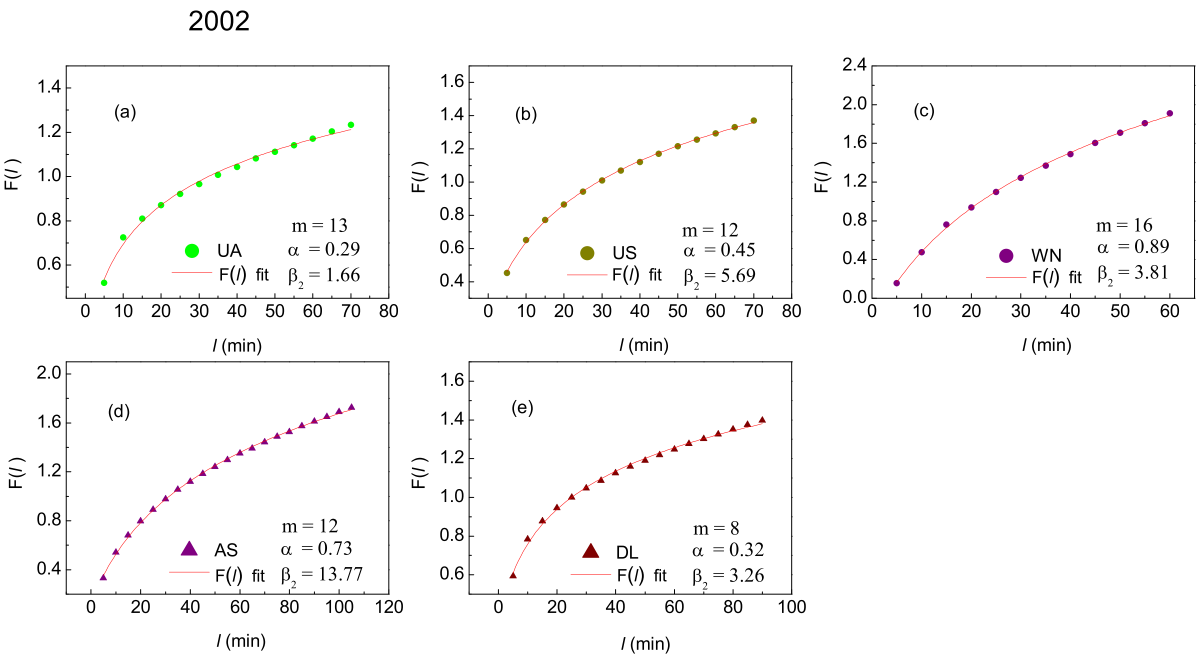

FIG. S47. $F(l) = \alpha ln(1 + \frac{l}{\beta_2})$(red lines) fitting cumulated transfer function mined from primary data of airline UA(a), US(b), WN(c), AS(d) and DL(e) in 2002. $\Delta l =$ *5min*.

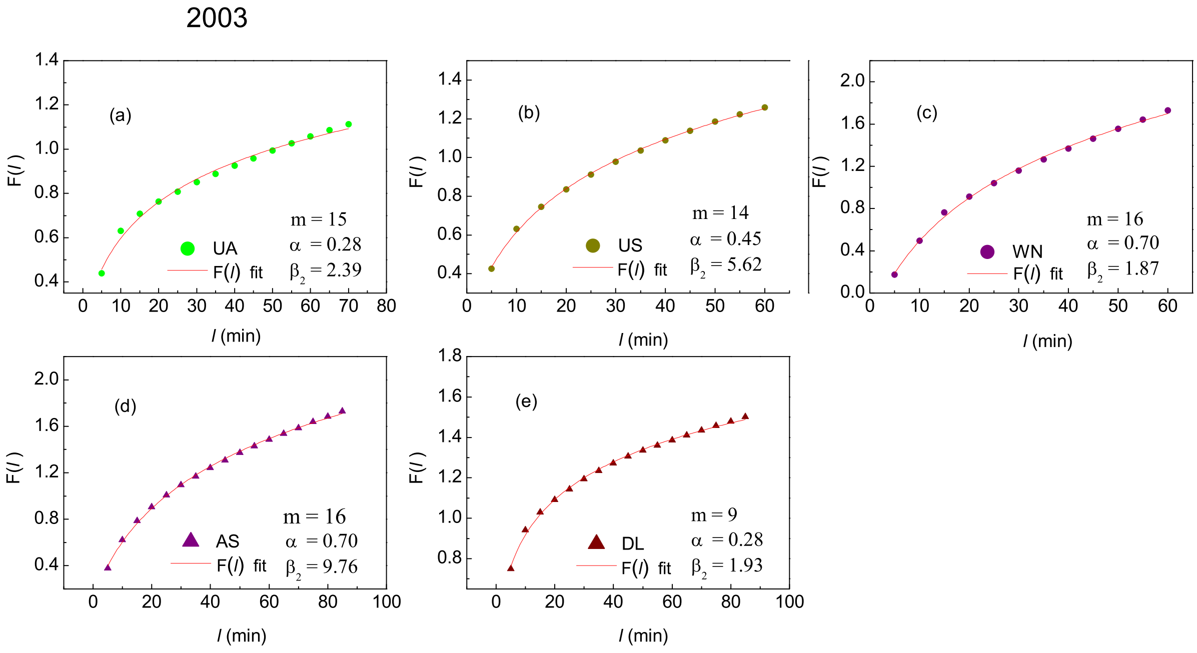

FIG. S48. $F(l) = \alpha ln(1 + \frac{l}{\beta_2})$(red lines) fitting cumulated transfer function mined from primary data of airline UA(a), US(b), WN(c), AS(d) and DL(e) in 2003. $\Delta l = 5min$.

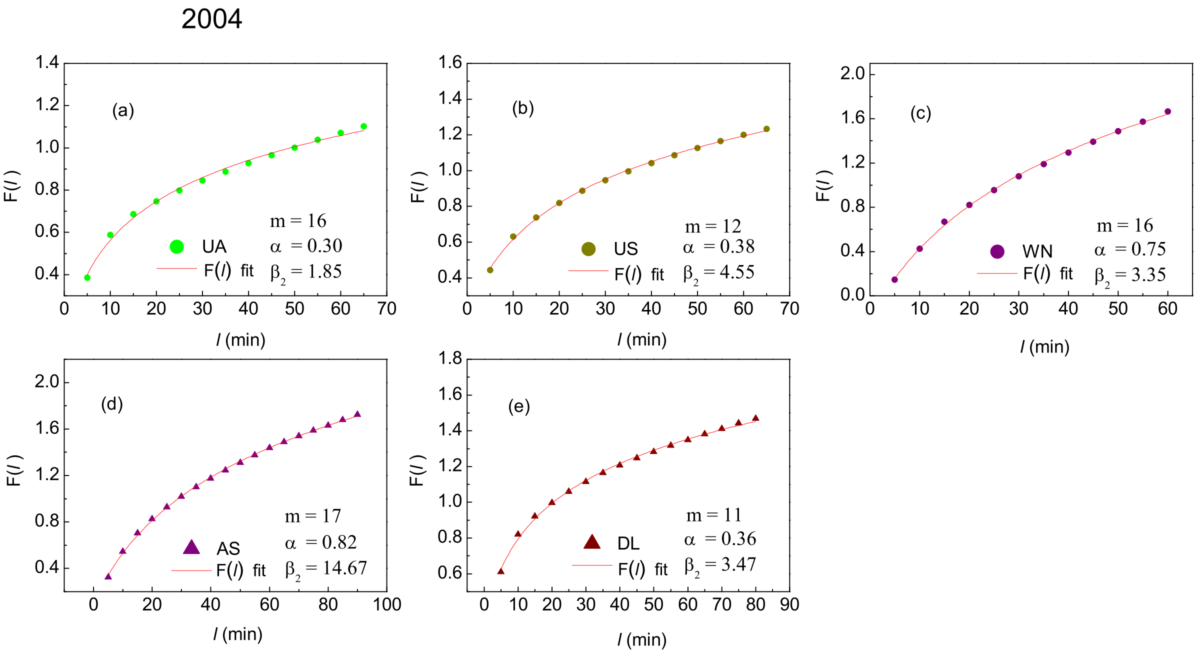

FIG. S49. $F(l) = \alpha ln(1 + \frac{l}{\beta_2})$(red lines) fitting cumulated transfer function mined from primary data of airline UA(a), US(b), WN(c), AS(d) and DL(e) in 2004. $\Delta l = 5min$.

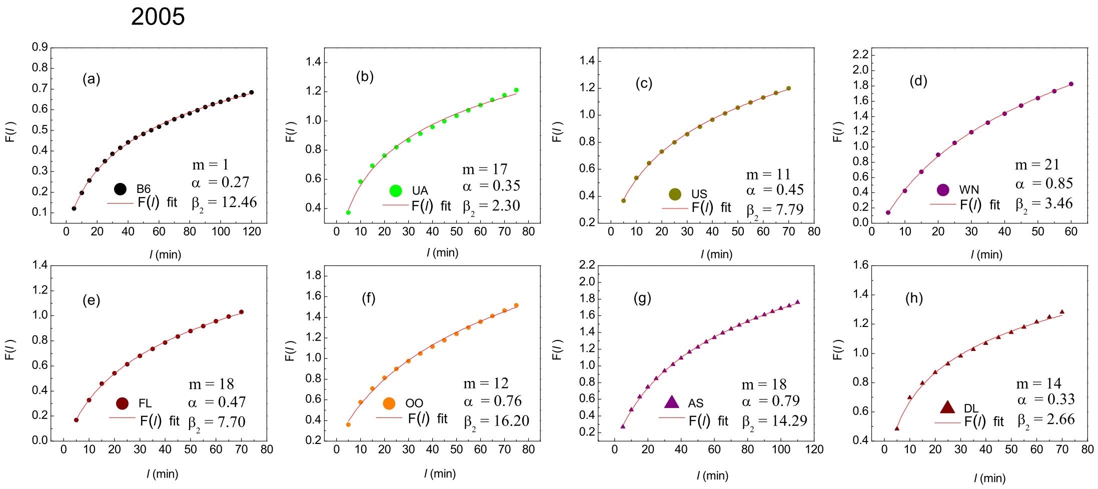

FIG. S50. $F(l) = \alpha ln(1 + \frac{l}{\beta_2})$(red lines) fitting cumulated transfer function mined from primary data of airline B6(a),UA(b), US(c), WN(d), FL(e), OO(f), AS(g) and DL(h) in 2005. $\Delta l = 5min$.

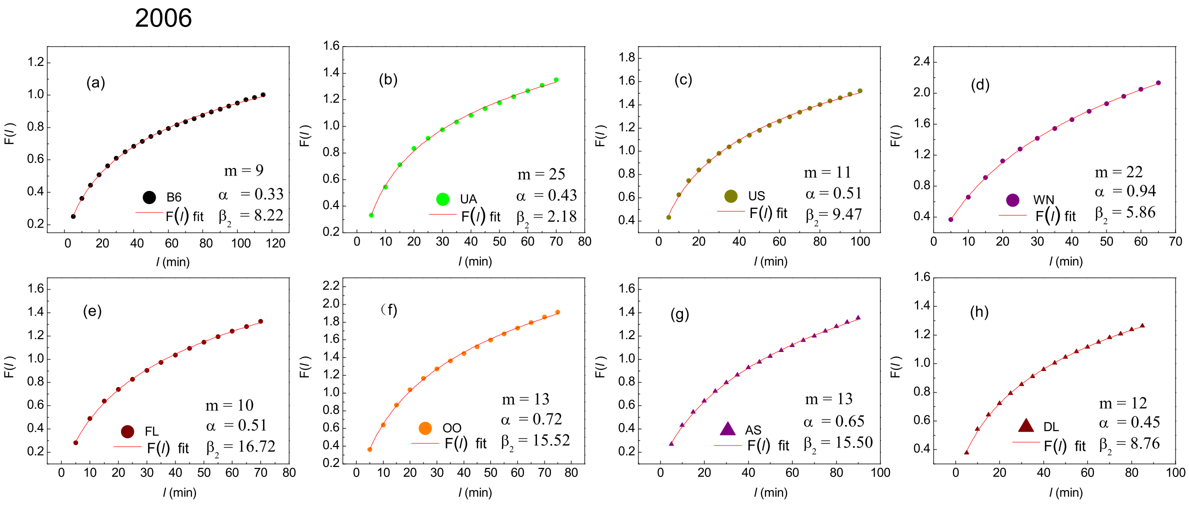

FIG. S51. $F(l) = \alpha ln(1 + \frac{l}{\beta_2})$(red lines) fitting cumulated transfer function mined from primary data of airline B6(a),UA(b), US(c), WN(d), FL(e), OO(f), AS(g) and DL(h) in 2006. $\Delta l = 5min$.

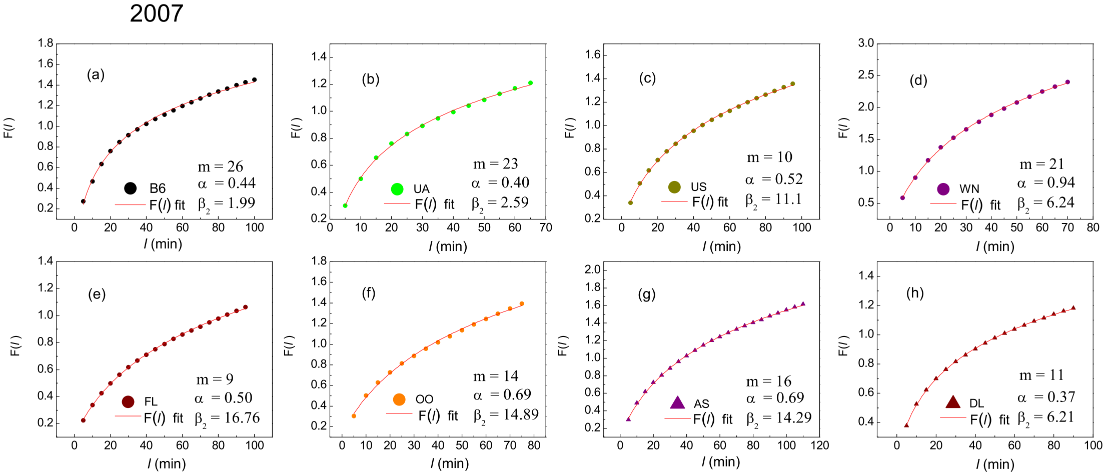

FIG. S52. $F(l) = \alpha ln(1 + \frac{l}{\beta_2})$(red lines) fitting cumulated transfer function mined from primary data of airline B6(a),UA(b), US(c), WN(d), FL(e), OO(f), AS(g) and DL(h) in 2007. $\Delta l = 5min$.

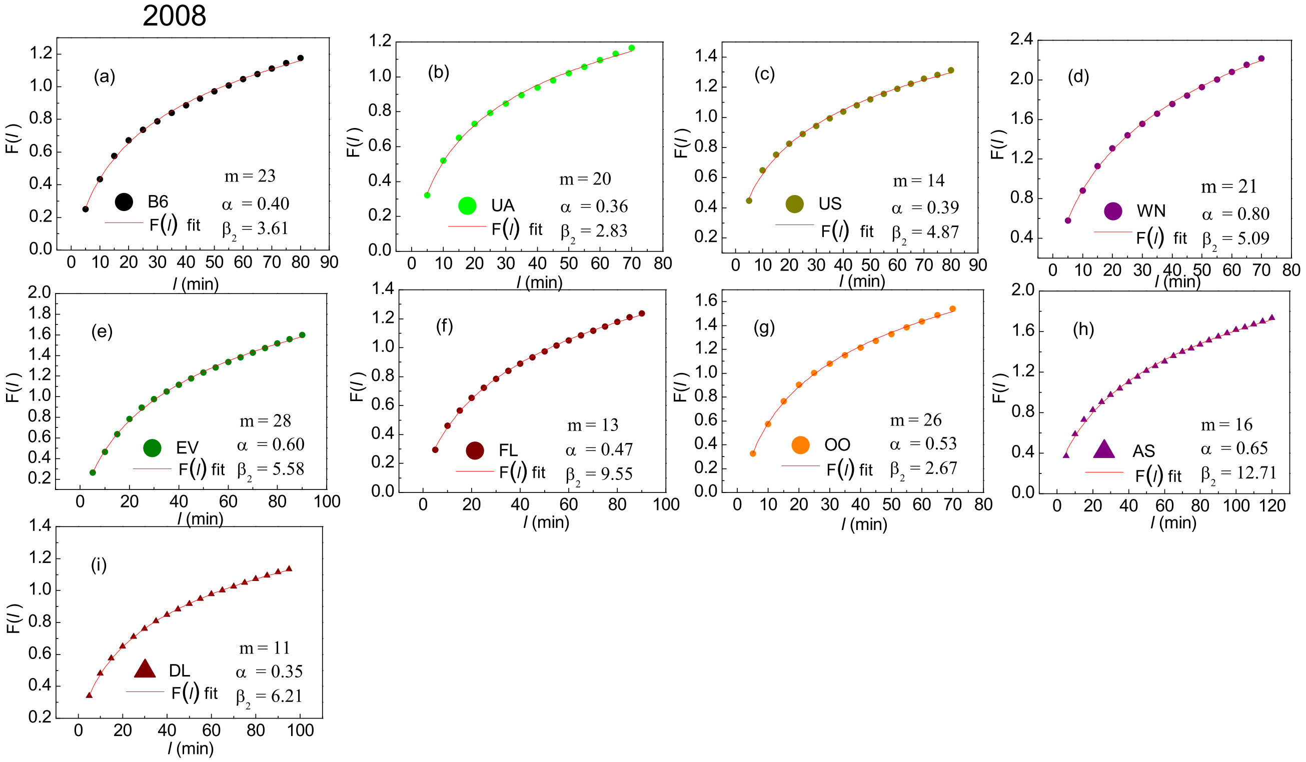

FIG. S53. $F(l) = \alpha ln(1 + \frac{l}{\beta_2})$(red lines) fitting cumulated transfer function mined from primary data of airline B6(a),UA(b), US(c), WN(d), EV(e), FL(f), OO(g), AS(h) and DL(i) in 2008. $\Delta l = 5min$.

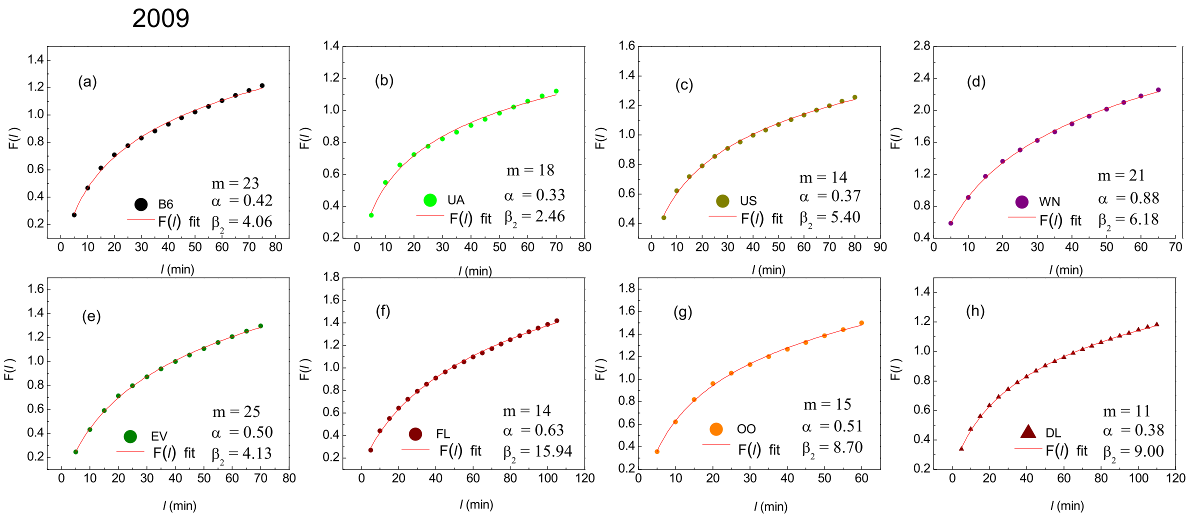

FIG. S54. $F(l) = \alpha ln(1 + \frac{l}{\beta_2})$(red lines) fitting cumulated transfer function mined from primary data of airline B6(a),UA(b), US(c), WN(d), EV(e), FL(f), OO(g) and DL(h) in 2009. $\Delta l = 5min$.

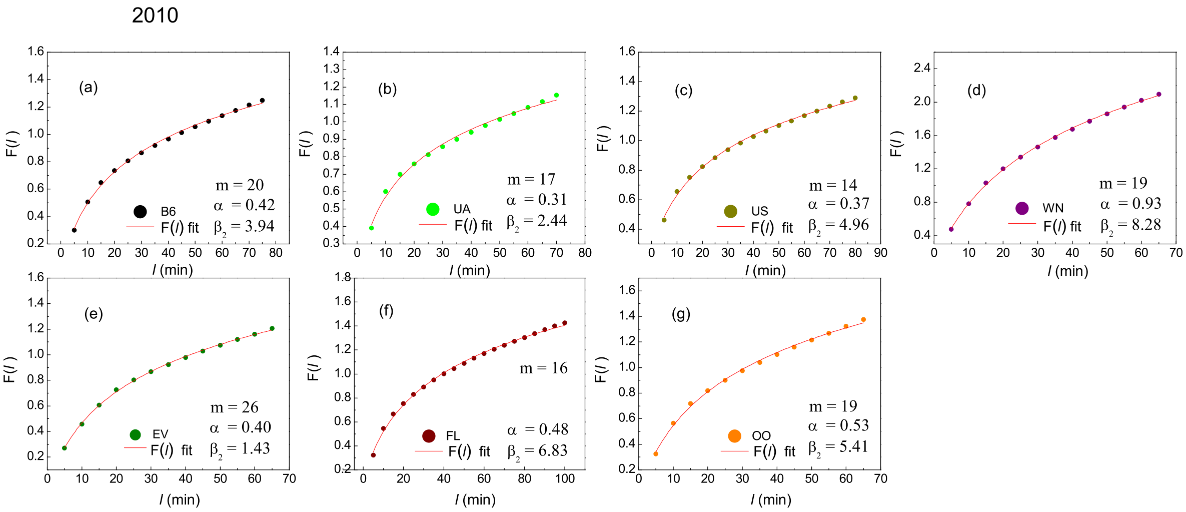

FIG. S55. Complementary cumulative distribution functions of departure delays l for airline B6(a),UA(b), US(c), WN(d), EV(e), FL(f) and OO(g) in 2010. $\Delta l = 5min$.

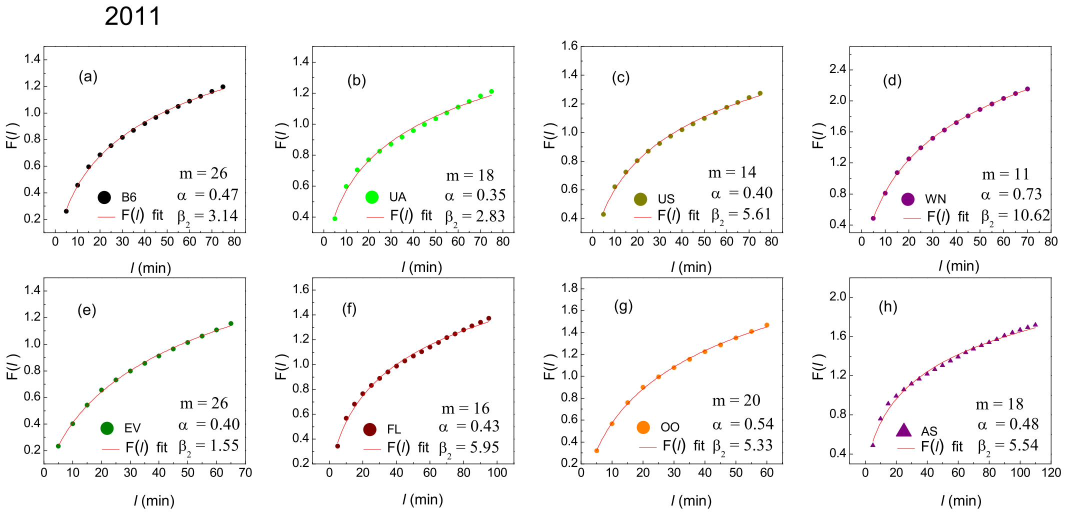

FIG. S56. Complementary cumulative distribution functions of departure delays l for airline B6(a),UA(b), US(c), WN(d), EV(e), FL(f), OO(g) and AS(h) in 2011. $\Delta l = 5min$.

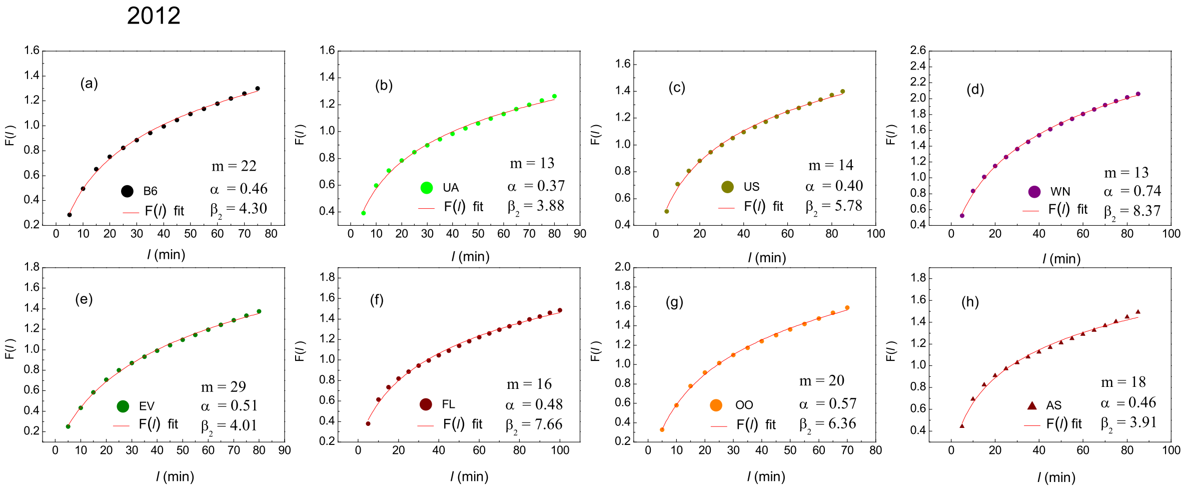

FIG. S57. Complementary cumulative distribution functions of departure delays l for airline B6(a),UA(b), US(c), WN(d), EV(e), FL(f), OO(g) and AS(h) in 2012. $\Delta l = 5min$.

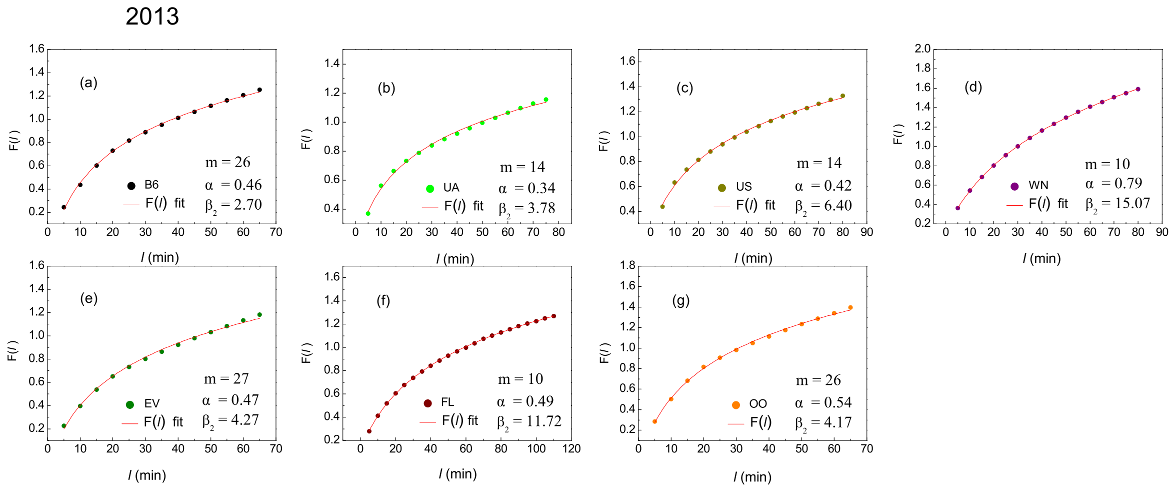

FIG. S58. Complementary cumulative distribution functions of departure delays l for airline B6(a),UA(b), US(c), WN(d), EV(e), FL(f) and OO(g) in 2013. $\Delta l = 5min$.

AVERAGE DELAY - ABSORBING TIME L VERSUS SHIFT QUANTITY β_1 IN EACH YEAR FOR AIRLINES IN BOTH GROUPS (FIG. S59 – FIG. S68)

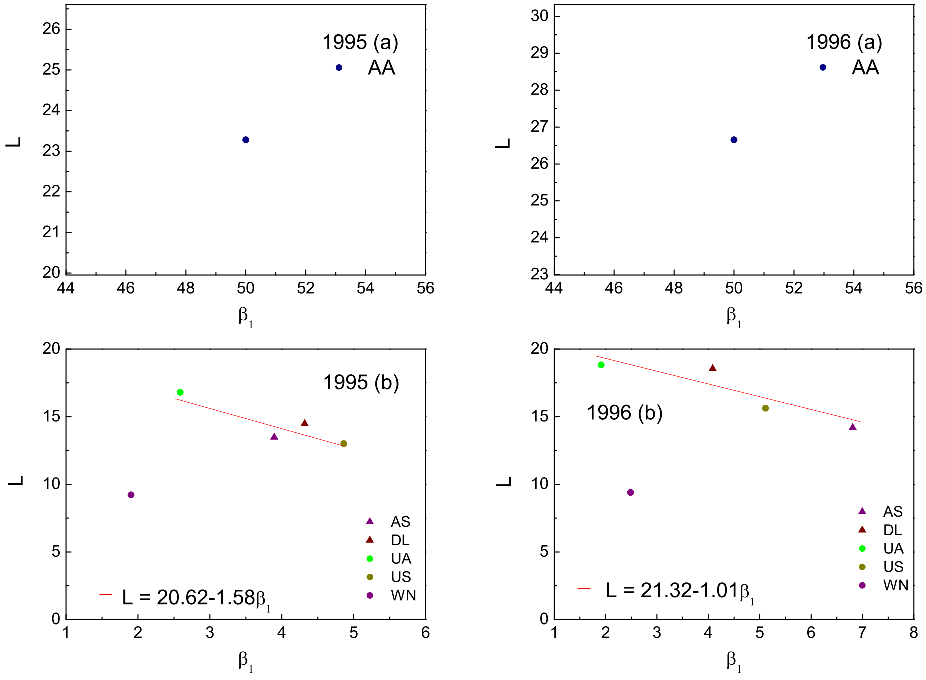

FIG. S59. Average delay - absorbing time versus shift quantity β_1 from 1995 to 1996. Upper panels(a): Group 1 with SPL; lower panels(b): Group 2 with ETSPL, for each year.

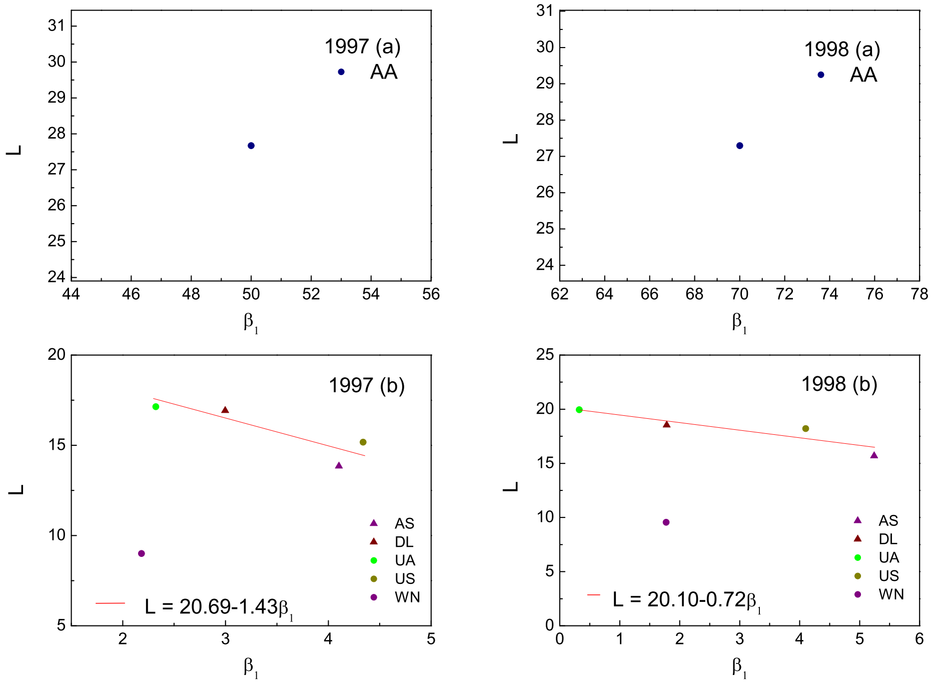

FIG. S60. Average delay - absorbing time versus shift quantity β_1 from 1997 to 1998. Upper panels(a): Group 1 with SPL; lower panels(b): Group 2 with ETSPL, for each year.

TEMPORAL VARIATIONS OF THREE METRICS DURING 20 YEARS(FIG. S69)

Temporal variations of three metrics — the shift quantity β_1, the compensation interval m, and the critical delay λ, reveal the performance evolution of the whole domestic passenger air transportation of the United States, and the operational features of individual airline companies. Besides all the figures for 2014, statistical results for the years 1995 $---$ 2013 are all displayed in **SI**: Fig. S1 — Fig. S10 and Fig. S11 — Fig. S29 for CCDFs in Group 1 and Group 2; Fig. S30 — Fig. S39 and Fig. S40 — Fig. S58 for $F(l)$ in Group 1 and Group 2; And Fig. S59 – Fig. S68 for relations $L(\beta_1)$ with all panels (a) for Group 1, all panels (b) for Group 2, respectively.

From 1995 to 2004, data of only six airlines are available. Group 1 contains only airline AA, while Group 2 contains five other airlines: UA, US, WN, AS and DL. After 2005, data from thirteen airlines are available. Airlines AA, MQ, F9, EV and HA stay in Group 1, while B6, UA, US, WN, FL, OO, AS and DL stay in Group 2, from 2005 to 2007. EV jumps to Group 2 in 2008 and stay there afterward. AS jumps to Group 1 in 2009, then jumps back in 2011, and jumps again to Group 1 in 2013. DL jumps to it in 2010 and stays there afterward. In the year 2014, airline VX appears in Group 2. Due to such jumps, temporal variation behaviors of β_1, m and λ interrupt at corresponding years as shown in Fig. S69.

As we argued in last section, β_1 in general measures inversely the ability of an airline to absorb the delay in the case of DTPD. From Fig. S66 – Fig. S68 we see that negative relations similar to formula (11) cover airlines in Group 1 from 2009 to 2013 except 2011, while those similar to formula (12) cover Group 2 for all 20 years. No correlation in Group 1 can be found in each panel with SPL before 2005, since only a representing point of airline AA appears. In the $L(\beta_1)$ graphs after 2004, points of HA keep staying at the positions with lower levels of both L and β_1. Its CCDF less deviates from a power-law meaning heterogeneity. In 2005 and 2006, points for airline F9 stay leftward, exhibiting more heterogeneity of their CCDFs. In addition, points for AA shift rightward in 2007 and 2008, exhibiting more homogeneity of their CCDFs. Their behaviors ruin the deterministic negative correlation between L and β_1 (see Fig. S64,Fig. S65 and Fig. S67). Actually, airlines MQ and EV (before its jumping in 2008) varies less than F9 and AA with either L or β_1 in the same panels. Moreover, in view of little transposition of points of HA which

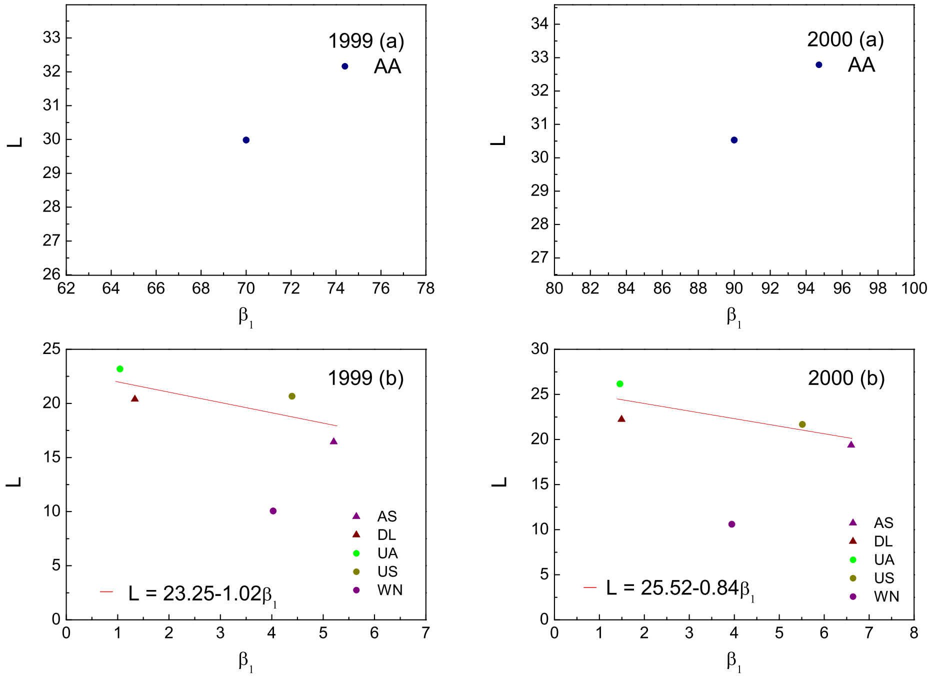

FIG. S61. Average delay - absorbing time versus shift quantity β_1 from 1999 to 2000. Upper panels(a): Group 1 with SPL; lower panels(b): Group 2 with ETSPL, for each year.

keep being isolated from the main strain line of statistical fitting in other years, no obvious negative correlation of $L(\beta_1)$ could be found in Group 1 during 2005 to 2008(also 2011). While the relations $L(\beta_1)$ of Group 2 behave in a negatively linear dependence except airline WN which does not follow the main strain relation and keeps staying at lower L (around 10 minutes) during 15 years (1995 – 2009). This is reminiscent of us with distinctive feature of its point-to-point operation strategy(which is also true for airline HA when only domestic flights are concerned). Detail investigation of such cases would be expected. It is noticeable that airline WN joins the main strain of negatively linear correlations $L(\beta_1)$ from 2010 (Fig. S66 – Fig. S68, and Fig.3**A** in the text), keeping its low delay - absorbing level but with increased β_1 from 2010 to 2014 in Group 2. Therefore, for majority of the airlines and for most years, a negative linear correlation between the average delay - absorbing L and β_1 is valid. We should conclude that, the more homogeneity of an airlines's DD distribution, the weaker ability for it to absorb immediate preceding delay with the same aircraft, in general.

In observation of the temporal variations(see Fig. S69 (a) and (b)), β_1 around 2005 – 2008 has higher or lower bumps compared with the previous values except B6 and UA, which means decreased absorption of delays by other 6 airlines. Simultaneous appearance of bumps of β_1 by AA, MQ, F9 and HA, especially F9 approaching HA, corresponds to the invalidation of negative linear correlation between L and β_1. After 2008, F9 (Fig. S69(a)) and WN (Fig. S69(b)) continue their essential increase of β_1. While after 2013, a new round of β_1-increase happen to almost all airlines (except the new comer VX), which could predict similar situation Group 2 faced during last bump of β_1. In contradiction, we can rank UA the best since it keeps lowest β_1 hence highest L in almost all years.

For airlines in group 2, FL, AS, US and OO have larger values β_1 from 2005 to 2008, which means decreased ability to absorb DTPD. More importantly, due to quicker drop to zero than the $k(l)$ assumed in Model 2, critical delay λ plays more important role than β_1 or β_2 in CCDFs. Usually, larger λ and smaller m enable higher precision of the phenomenological models (see (c), (d), (e) and (g) of Fig.2**A** and Fig.2**B** in the text, for examples). Large m strongly reduces the effect of DTPD assumed in formula (1). Airlines EV and B6 after 2008 (see panels (a) and (e) of Fig. S27 — Fig. S31, Fig. S55 — Fig. S60, UA and WN during 2005 — 2008 (see (b) and (d) of Fig. S23 — Fig. S27, Fig. S52 — Fig. S56), and OO in 2008 and 2013 (see panels (g) of Fig. S26 — Fig. S31, Fig. S55 — Fig. S60, respectively,

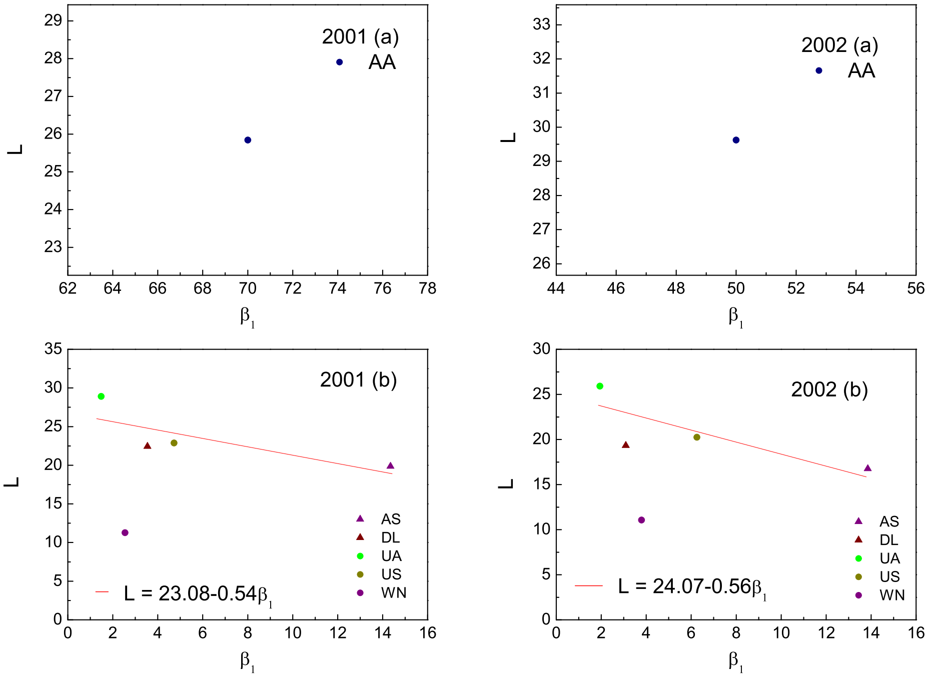

FIG. S62. Average delay - absorbing time versus shift quantity β_1 from 2001 to 2002. Upper panels(a): Group 1 with SPL; lower panels(b): Group 2 with ETSPL.

serve the worse examples than other airlines during the same years, and in comparison with their own performance in other years.

Comparing panel (b) with (c) in Fig. S69, one can easily see that the levels of λ are always higher than corresponding β_1 in Group 2, and λ sometime share similar oscillating-increase tendency of β_1, say, airlines UA, WN, B6 US, OO, and EV, for examples. Observation of the λ - variation can further divide the operation behaviors of the airlines into 4 subgroups: AS, FL and DL often oscillating at higher level above 80; UA, US and WN having gentle oscillations and increases often below 80; B6, EV, and OO having continuous valleys; and finally VX, the isolated new comer. Common behaviors within individual subgroups and difference between them characterized by both β_1 and λ are expected to be understood by investigation to detailed operations.

In addition to the λ - valleys they have, airlines B6, EV and OO have higher levels of m(see Fig. S69(c) and (d)), which means more chances to be affected by NPF and ITPD. Besides, UA and WN both undergo m - bumps after 2005, then decrease essentially after 2008, which indicates the decrease of effect of NPF and ITPD and growing operation order by DTPD from 2009. Moreover, both WN and US display stable levels of m for a few years, which indicates the operational stability of the airlines. Finally, m of all airlines drop down simultaneously from 2013, indicating the growing tendency of more DTPD in the flight delay events.

* These two authors contributed equally
† To whom correspondence may be addressed. Email: oldpigman1234@126.com, hes@bu.edu

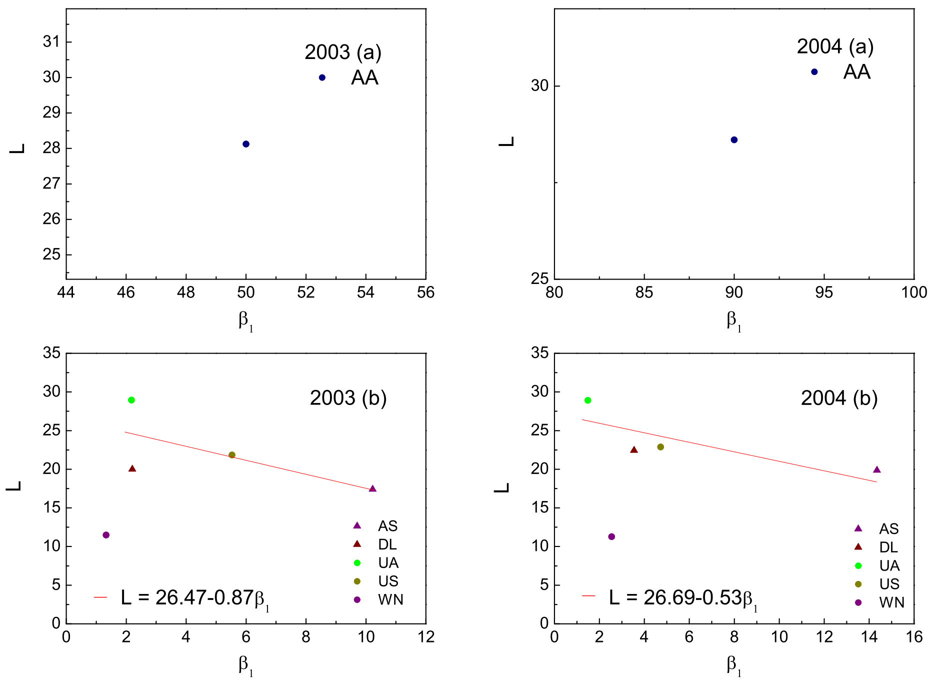

FIG. S63. Average delay - absorbing time versus shift quantity β_1 from 2003 to 2004. Upper panels(a): Group 1 with SPL; lower panels(b): Group 2 with ETSPL, for each year.

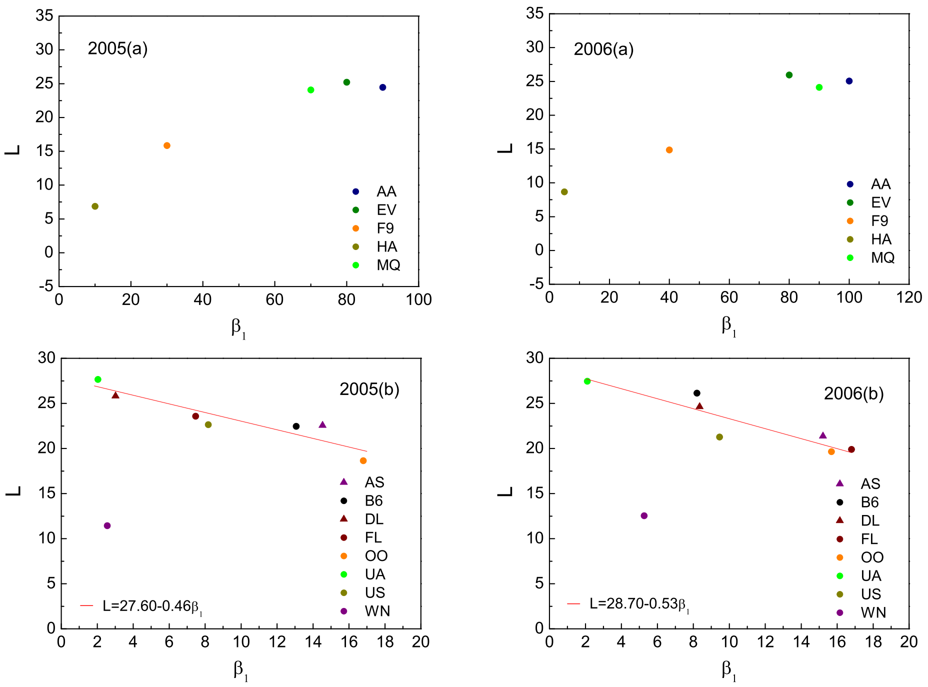

FIG. S64. Average delay - absorbing time versus shift quantity β_1 from 2005 to 2006. Upper panels(a): Group 1 with SPL; lower panels(b): Group 2 with ETSPL.

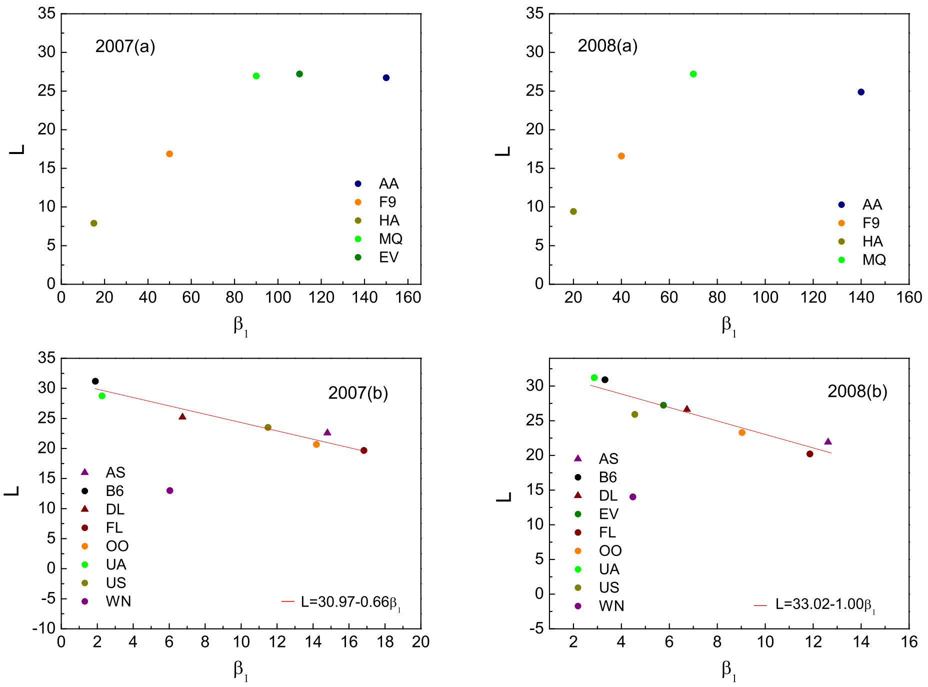

FIG. S65. Average delay - absorbing time versus shift quantity β_1 from 2007 to 2008. Upper panels(a): Group 1 with SPL; lower panels(b): Group 2 with ETSPL, for each year.

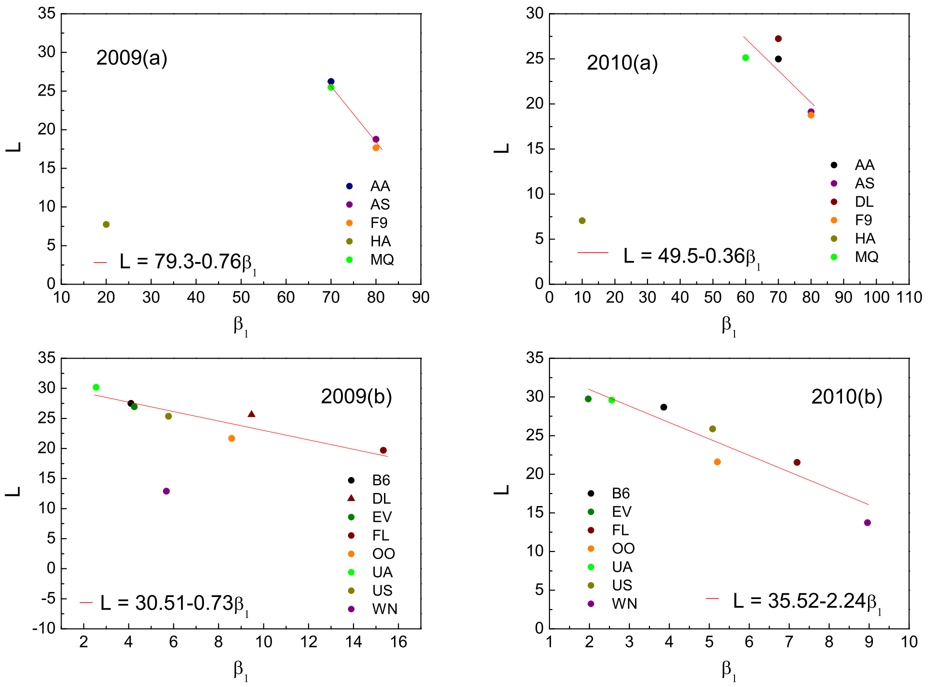

FIG. S66. Average delay - absorbing time versus shift quantity β_1 from 2009 to 2010. Upper panels(a): Group 1 with SPL; lower panels(a): Group 2 with ETSPL.

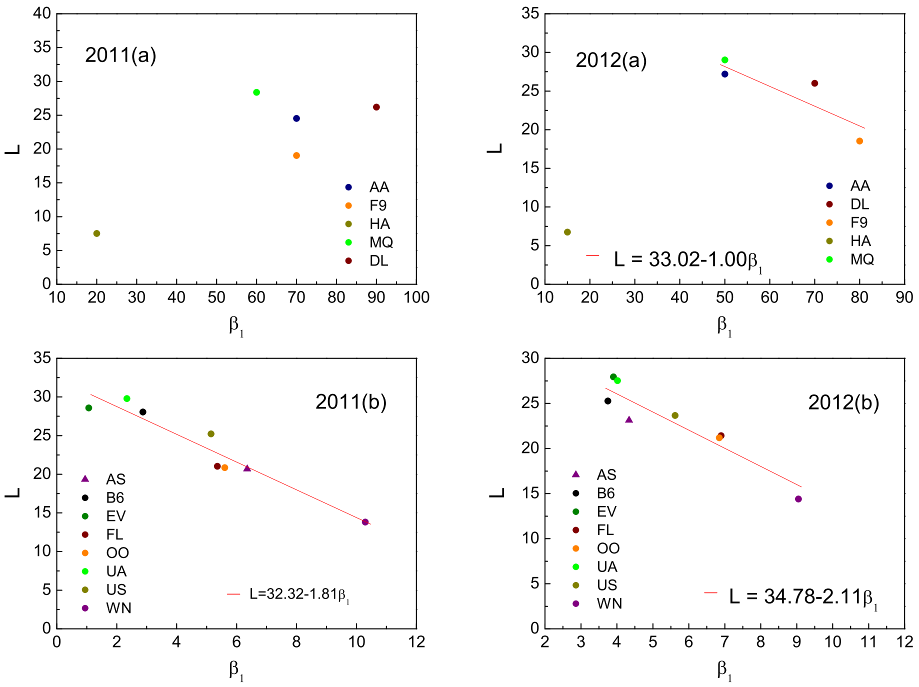

FIG. S67. Average delay - absorbing time versus shift quantity β_1 from 2011 to 2012. Upper panels(a): Group 1 with SPL; lower panels(b): Group 2 with ETSPL.

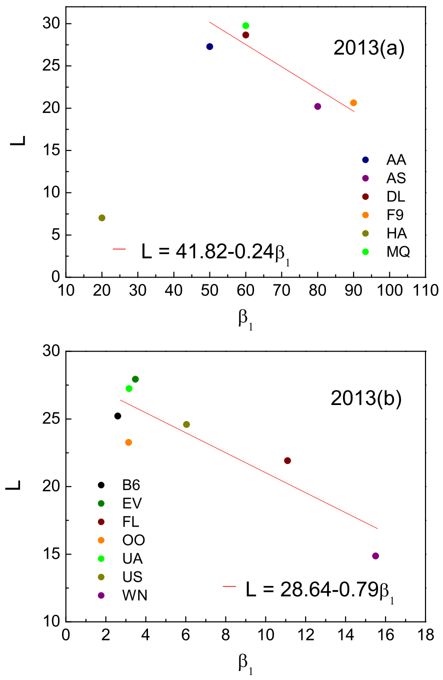

FIG. S68. Average delay - absorbing time versus shift quantity β_1 in 2013. Upper panels(a): Group 1 with SPL; lower panels(b): Group 2 with ETSPL.

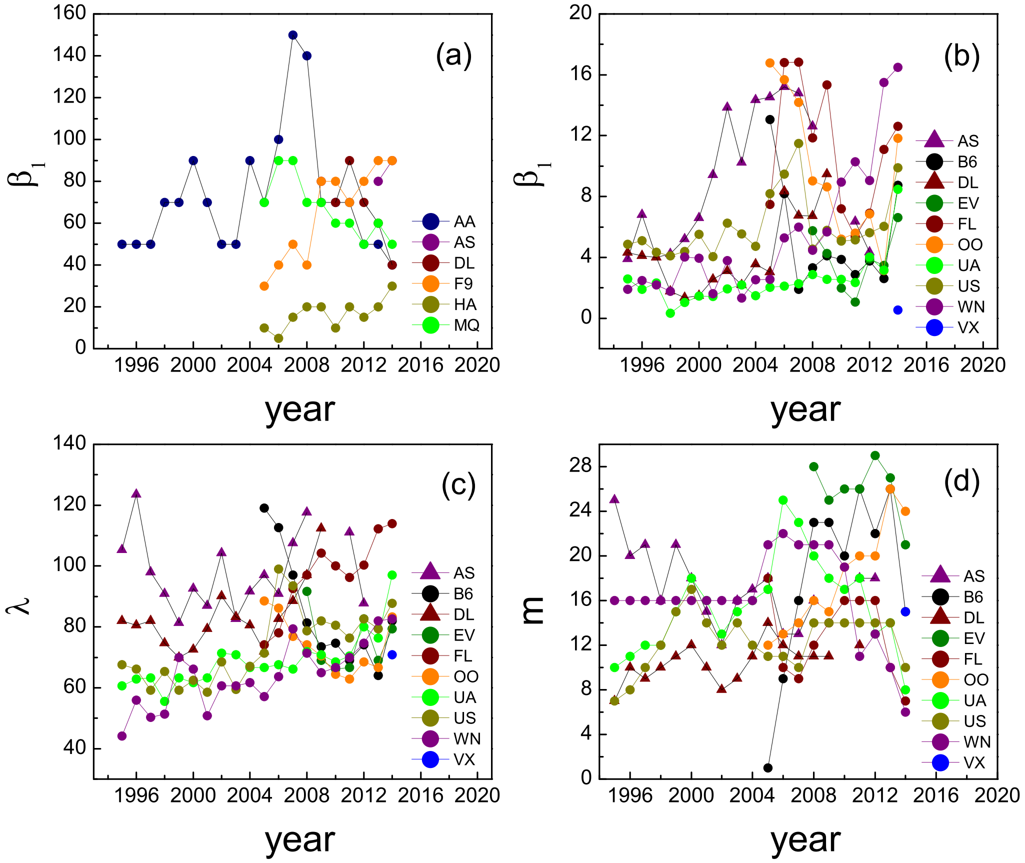

FIG. S69. Temporal variations of three metrics from 1995 to 2014. (a) Shift quantity $\beta_1(t)$ for airlines in Group 1. (b) Shift quantity $\beta_1(t)$ for airlines in Group 2. (c) Critical delay $\lambda(t)$ for Group 2 with ETSPL. (d)Variations of compensation intervals $m(t)$ with time for Group 2 with ETSPL.
